# The uncoordinated‐5 homologue A is a key receptor in netrin‐ligand‐mediated fast‐twitch myotube formation in male mice

**DOI:** 10.14814/phy2.70788

**Published:** 2026-02-17

**Authors:** Takahiro Maeno, Tomoki Ushijima, Koichi Ojima, Yohei Ogawa, Sayuki Hayashi, Hikaru Imakyure, Rika Osaki, Ryuki Oyama, Aoi Ogawa, Akimasa Takano, Kaoru Mizoguchi, Issei Yokoyama, Yusuke Komiya, Mako Nakamura, Ryuichi Tatsumi, Takahiro Suzuki

**Affiliations:** ^1^ Laboratory of Muscle and Meat Science, Department of Animal and Marine Bioresource Sciences, Research Faculty of Agriculture, Graduate School of Agriculture Kyushu University Fukuoka Japan; ^2^ Muscle Biology Research Unit, Division of Animal Products Research Institute of Livestock and Grassland Science, NARO Tsukuba Japan; ^3^ Department of Animal Science, School of Veterinary Medicine Kitasato University Towada Japan; ^4^ Present address: Department of Muscle Development and Regeneration, Institute of Molecular Embryology and Genetics (IMEG) Kumamoto University Kumamoto Japan

**Keywords:** fast‐twitch myotube, myofiber type, myotube, netrin, satellite cell‐derived myoblast, uncoordinated‐5 homologue A

## Abstract

Myoblasts autonomously govern myofiber‐type specification of newly formed myotubes through autocrine–paracrine‐dependent manners mediated by multipotent modulators. Netrin‐1, which is particularly produced in myoblasts isolated from the extensor digitorum longus (EDL; fast‐twitch myofiber‐abundant) rather than the soleus (slow‐twitch myofiber‐abundant), and netrin‐4, which is abundantly expressed during myogenic differentiation initiation, stimulate the synthesis of fast‐type myosin heavy chain (MyHC) isoforms. However, the mechanisms by which netrin‐1 and netrin‐4 promote fast‐twitch myotube formation remain unclear. Here, we investigated the roles of netrin receptors, uncoordinated‐5 homologues (UNC5A, ‐B, ‐C, and ‐D), deleted in colorectal cancer (DCC), and the DCC paralog (neogenin) during myogenic differentiation, focusing on fast‐twitch myotube formation. We confirmed that UNC5A, UNC5B, UNC5C, and neogenin synthesis patterns in EDL myoblasts showed no marked differences compared with those in soleus myoblasts. Notably, UNC5A knockdown severely inhibited fast‐twitch myotube formation compared with other receptor knockdown treatments and significantly reduced the synthesis of fast‐type MyHC isoforms. Additional treatment with recombinant netrin‐1 or netrin‐4 induced fast‐type MyHC mRNA expression; however, this effect was suppressed by UNC5A knockdown. These findings revealed that UNC5A is involved in fast‐twitch myotube formation via netrin ligands, highlighting an autonomous fast‐type myofiber commitment system within myoblasts.

## Graphical abstract

Unc5A, a netrin ligand‐receptor expressed in satellite cell‐derived myoblasts, may regulate the formation of fast‐twitch myotubes by stimulating fast‐type myosin heavy chain synthesis.

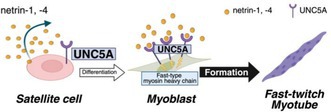

## 1 INTRODUCTION

Skeletal muscles are collectively the heaviest tissue in the mammalian body and are crucial for posture, voluntary movements, respiration, and metabolic functions. A muscle is composed of thousands of bundled multinucleated myofibers and exhibits an exceptional capacity for fully recovering structural and contractile functions following injury (Sousa‐Victor et al., 2022). Myoregeneration and hypertrophy are mainly mediated by satellite cells (myogenic stem cells) located beneath the basal lamina. Upon injury‐induced activation, satellite cells proliferate and differentiate into myoblasts, which fuse to damaged areas of myofibers or newly form multinucleated myotubes (Hindi et al., 2013; Relaix et al., 2021).

Myofibers are classified into slow‐twitch [myosin heavy chain (MyHC) type I‐positive] and fast‐twitch (MyHC type II‐positive) fibers, with fast‐twitch fibers further subdivided into type IIa, IIx, and IIb‐positive fibers. Type IIa represents fast‐twitch oxidative fibers; type IIx, intermediate fibers; and type IIb, fast‐twitch glycolytic fibers. Each type possesses distinct contraction speeds, metabolic characteristics, and fatigue resistance levels that influence muscle performance (Schiaffino & Reggiani, 2011). Myofiber type is influenced by neural stimulation patterns, mechanical loading, hormone activity, nutrition, genetic determinants, and pathological conditions such as cancer cachexia and diabetes (Blaauw et al., 2013; Ciciliot et al., 2013). Intriguingly, satellite cells from different muscle types have a unique differentiation potential that autonomously regulates myofiber‐type specification (Motohashi et al., 2019). Those isolated from slow‐twitch muscles, such as the soleus, are more likely to form slow‐twitch myotubes expressing MyHC type I, whereas those isolated from fast‐twitch muscles, such as the extensor digitorum longus (EDL) and tibialis anterior (TA), tend to develop into fast‐twitch myotubes expressing MyHC type II (Furuichi et al., 2021; Lagord et al., 1998; Maeno et al., 2023; Rosenblatt et al., 1996).

To elucidate how the myofiber type of myotubes reflects the characteristics of the skeletal muscle from which myoblasts were originally derived, we focused on several reports suggesting that the secreted factors regulate myogenic events in an autocrine‐paracrine‐dependent manner, including multipotent molecules such as semaphorins, netrins, ephrins and slits (Cho et al., 2021; Siegel et al., 2009; Stark et al., 2015; Tatsumi et al., 2009). We also previously reported that semaphorin 3A, which is abundantly secreted by satellite cells derived from the soleus muscles, promotes the formation of slow‐twitch myotubes through autocrine/paracrine actions via its receptor neuropilin 2‐plexin A3 (Suzuki et al., 2013; Tatsumi et al., 2017). Netrin‐1, which is highly synthesized in myoblasts derived from the EDL muscles, contributes to fast‐twitch myotube formation via autocrine/paracrine mechanisms (Suzuki et al., 2021). Netrin‐4 promotes myoblast fusion and the expression of fast‐twitch MyHCs during myogenic differentiation (Maeno et al., 2023). These secreted netrin‐ligands directly bind to receptors such as deleted in colorectal cancer (DCC), the DCC paralog (neogenin), and uncoordinated‐5 homologues (UNC5A–D) in various types of cells (Sun et al., 2011). The previous reports showed that neogenin, UNC5B, and UNC5C are expressed in myoblasts, and neogenin has been shown to promote myogenic differentiation (Bae et al., 2009; do Carmo Costa et al., 2021; Kang et al., 2004; Suzuki et al., 2021). However, it remains unclear whether netrin receptors contribute to the autonomous myofiber type regulatory systems in satellite cell‐derived myoblasts.

Therefore, the purpose of this study was to investigate the roles of myoblast netrin receptors in myofiber type regulation of myotubes, particularly focusing on fast‐twitch myotube formation, such as netrin‐1 and netrin‐4 ligands. Neogenin, UNC5A, UNC5B, and UNC5C were stably synthesized during myoblast differentiation, with UNC5A showing increased expression. UNC5A, UNC5B, and UNC5C contributed to myotube formation, as well as neogenin. siRNA‐mediated knockdown of UNC5A revealed its key role in netrin‐induced fast‐twitch myotube formation.

## 2 MATERIALS AND METHODS

### 2.1 Animals

Animal studies were performed according to the Guidelines for Proper Conduct of Animal Experiments set forth by the Science Council of Japan and approved by the Kyushu University Institutional Review Board (approval numbers: A21‐039, A23‐210). 8‐week‐old male C57BL/6J mice (The Jackson Laboratory Japan, Kanagawa, Japan) were housed in a controlled environment at 23°C ± 2°C and 55% ± 10% humidity on a 12‐h light/dark cycle (lights on at 8 a.m.) with unrestricted access to food (CRF‐1, Oriental Yeast, Tokyo, Japan) and water.

### 2.2 Satellite cell isolation and single subculture; soleus or EDL myoblast cultures

**FIGURE 1:**
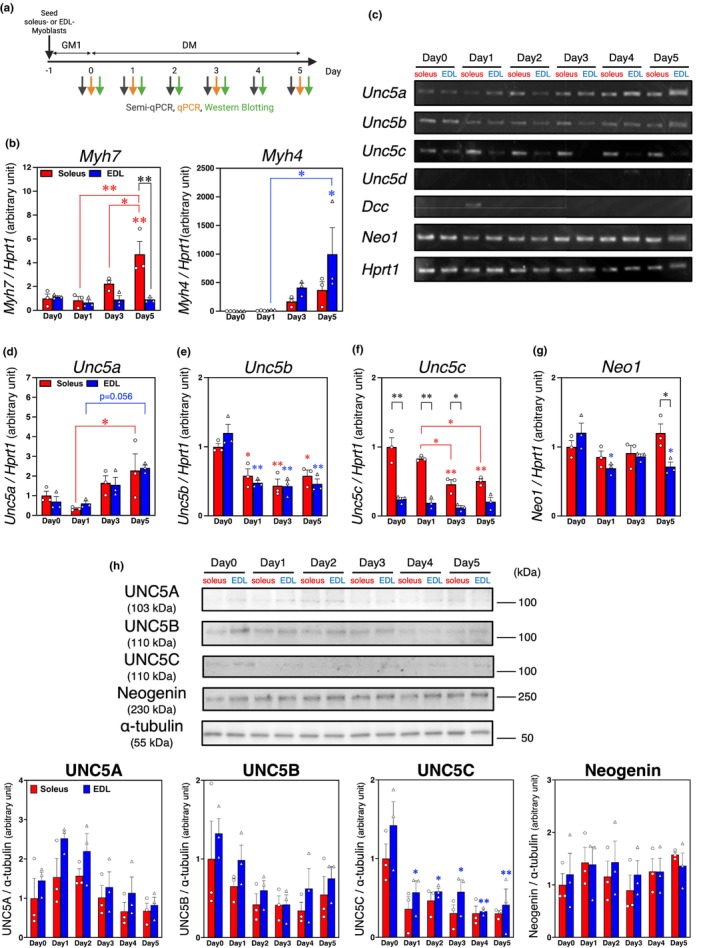
The expression and synthesis profile of UNC5A, UNC5B, UNC5C, and neogenin during myogenic differentiation in both soleus and EDL myoblasts. (a) Time course of differentiation induction. (b, d–g) qPCR analysis of myosin heavy chain (MyHC) isoform ([b]*Myh7* and *Myh4*) and netrin receptor ([d]*Unc5a*, [e]*Unc5b*, [f]*Unc5c*, and [g]*Neo1*) mRNA expression 0, 1, 3, and 5 days after induction of myogenic differentiation; *HPRT1* expression was used as an internal control. (c) Semi‐qPCR analysis, standardized to *HPRT1* expression, showing mRNA levels of *Unc5a*, *Unc5b*, *Unc5c*, *Unc5d*, *Neo1*, and *Dcc* 0–5 days after differentiation induction. (h) Western Blotting analysis of netrin receptor protein expression 0–5 days after induction, with α‐tubulin used as a loading control. The graph shows the quantification of band intensities normalized to α‐tubulin. The uncropped images of Western Blotting analysis are provided as Figure S1 ([S1A]UNC5A, [S1B]UNC5B, [S1C]UNC5C, [S1D]Neogenin, and [S1E]α‐tubulin). The bars depict the arbitrary units (means ± SEMs of three independent cultures) compared with soleus‐derived myoblast cultures on Day 0 post‐differentiation. Statistical comparisons were conducted within each myoblast type (soleus and EDL), and bars and asterisks indicated by red and blue represent soleus and EDL myoblasts, respectively. Significant differences at *p* < 0.05 and *p* < 0.01 are indicated by single and double asterisks, respectively (one‐way ANOVA followed by Tukey–Kramer post‐hoc test; *n* = 3).

Satellite cells were isolated as previously described (Maeno et al., 2023; Suzuki et al., 2025). Soleus and EDL muscles were dissected from mice and treated with 0.5% (*w/v*) type I collagenase (cat. no. LS004196, Worthington Biochemical, Freehold, NJ, USA) in Dulbecco's modified Eagle's medium (DMEM; cat. no. 0845816, Nacalai Tesque, Kyoto, Japan) at 37°C for 2 h (EDL) or 2.5 h (soleus). The muscles were transferred to 60‐mm Petri dishes coated with horse serum (HS; cat. no. SH30074, Hyclone, Cytiva, Tokyo, Japan) containing DMEM supplemented with 0.5% gentamicin (cat. no. 15710072, Gibco, Grand Island, NY, USA) and 1% antibiotic–antimycotic solution (AA‐mix; cat. no. 15240062, Gibco) using a heat‐polished HS‐coated Pasteur pipette. After repeated trituration to separate individual myofibers, incubation was performed overnight at 37°C. The liberated single myofibers were collected and placed into poly‐L‐lysine‐coated 6‐well multi‐plates (cat. no. 4810‐040, IWAKI, Asahi Glass, Tokyo, Japan) coated with 1 mg/mL Matrigel Basement Membrane Mix (cat. no. 356234, Corning, Bedford, MA, USA) in Growth Medium 1 [GM1; DMEM containing 30% fetal bovine serum (FBS; cat. no. 35‐079‐CV, Corning), 0.5% gentamicin reagent (Gibco), 1% AA‐mix (Gibco), and 2.5 ng/mL recombinant basic fibroblast growth factors (FGF2; cat. no. 3339‐FB‐025, R&D Systems, Minneapolis, MN, USA)] for 3–4 days. Myofibers adhering to the plate were removed, and only satellite cells that had migrated from the myofibers were maintained in GM1 for another 3–4 days. Proliferating satellite cells were detached using 0.05% trypsin–EDTA (Gibco) in pH −7.2 phosphate‐buffered saline (PBS) and reseeded onto poly L‐lysine‐coated 12‐ or 24‐well plates (cat. no. 4815‐040 or 4820‐040, IWAKI) containing 1 mg/mL Matrigel Basement Membrane Mix; 12‐ and 24‐well plates were seeded with 5.0 × 10^4^ and 2.5 × 10^4^ cells/well for protein and mRNA assays, respectively. After maintenance in GM1 for 1 day, satellite cells were transferred to differentiation medium (DM; DMEM with 5% HS, 0.5% gentamicin, and 1% AA‐mix) to promote myogenic differentiation and myotube formation for 1–5 days (Figure 1a). For RNA interference experiments, the medium was replaced with siRNA‐containing DM (Figures 4a and 5a). All cultures were maintained in a humidified atmosphere with 5% CO_2_ at 37°C.

### 2.3 Satellite cell‐derived myoblast culture

**FIGURE 2:**
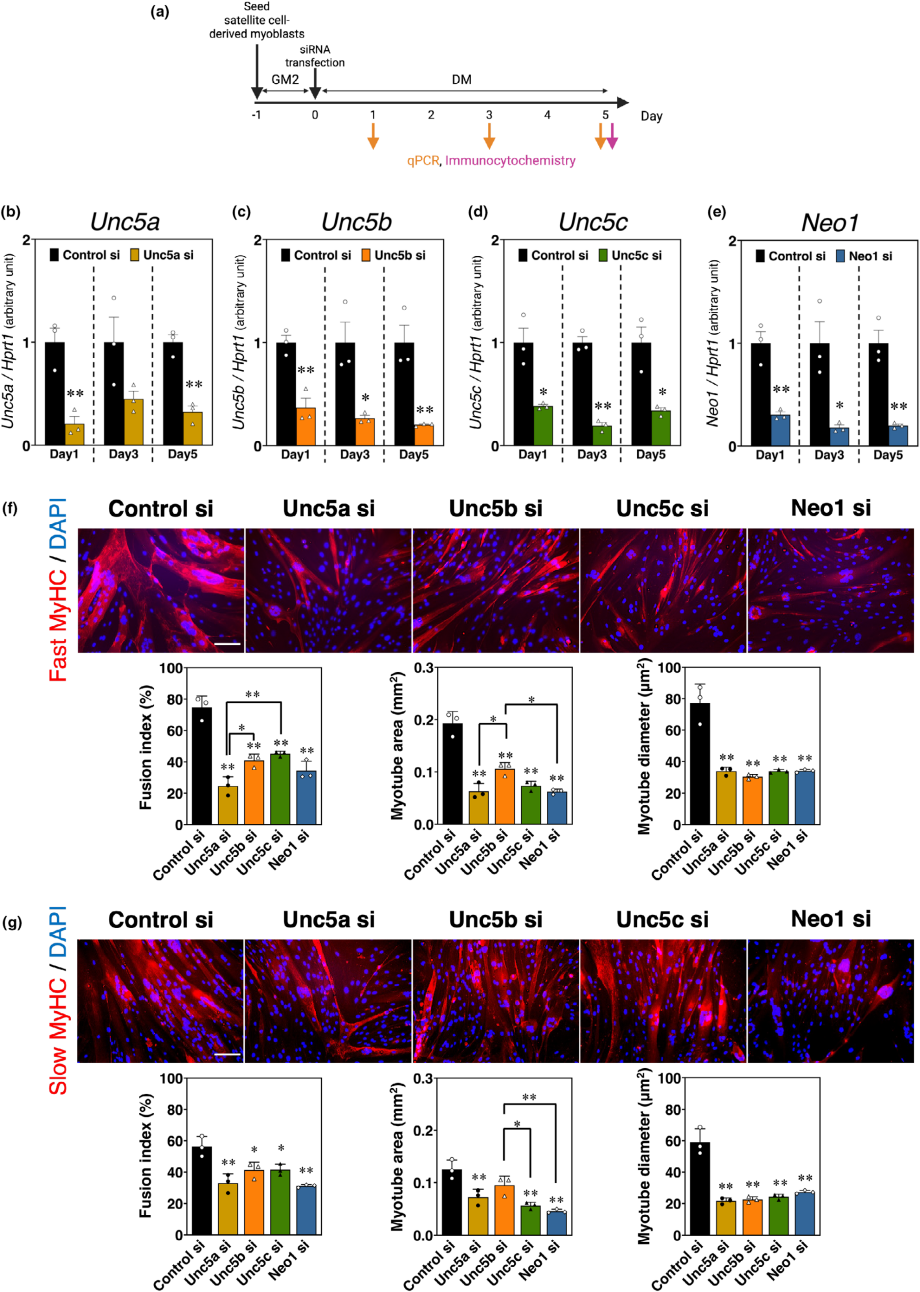
Suppressing UNC5A, UNC5B, UNC5C, or neogenin mRNA expression affected the formation of fast‐ and slow‐type myosin heavy chain positive‐myotubes. (a) Time course of UNC5A, UNC5B, UNC5C, or neogenin knockdown. (b–e) Netrin receptor mRNA expression in myoblasts with (b) UNC5A, (c) UNC5B, (d) UNC5C, or (e) neogenin knockdown on Days 1, 3, or 5 of myogenic differentiation was evaluated by qPCR analysis standardized to *HPRT1*. The bars, with each experimental group represented by a distinct color (Unc5a si: Yellow ochre; Unc5b si: Orange; Unc5c si: Green; Neo1 si: Blue), depict arbitrary units (means ± SEMs of three independent cultures) relative to control siRNA‐treated myoblast cultures (the black bar). Statistical comparisons were conducted within each time point. Significant differences from the control siRNA group mean at *p* < 0.05 and *p* < 0.01 are indicated by single and double asterisks, respectively (Student's *t*‐test; *n* = 3). (f and g) Immunocytochemistry for fast MyHC (red), slow MyHC (red), and nuclei (blue) in myoblasts with knockdown of UNC5A, UNC5B, UNC5C, or neogenin after 5 days of myogenic differentiation. Fusion index, Myotube area, and Myotube diameter were measured using Myotube Analyzer software. The results are expressed as means ± SEMs of three independent cultures. Significant differences from the means of the control siRNA group at *p* < 0.05 and *p* < 0.01 are indicated by single and double asterisks, respectively (one‐way ANOVA followed by Tukey–Kramer post‐hoc test; *n* = 3; scale bar = 100 μm).

**FIGURE 3:**
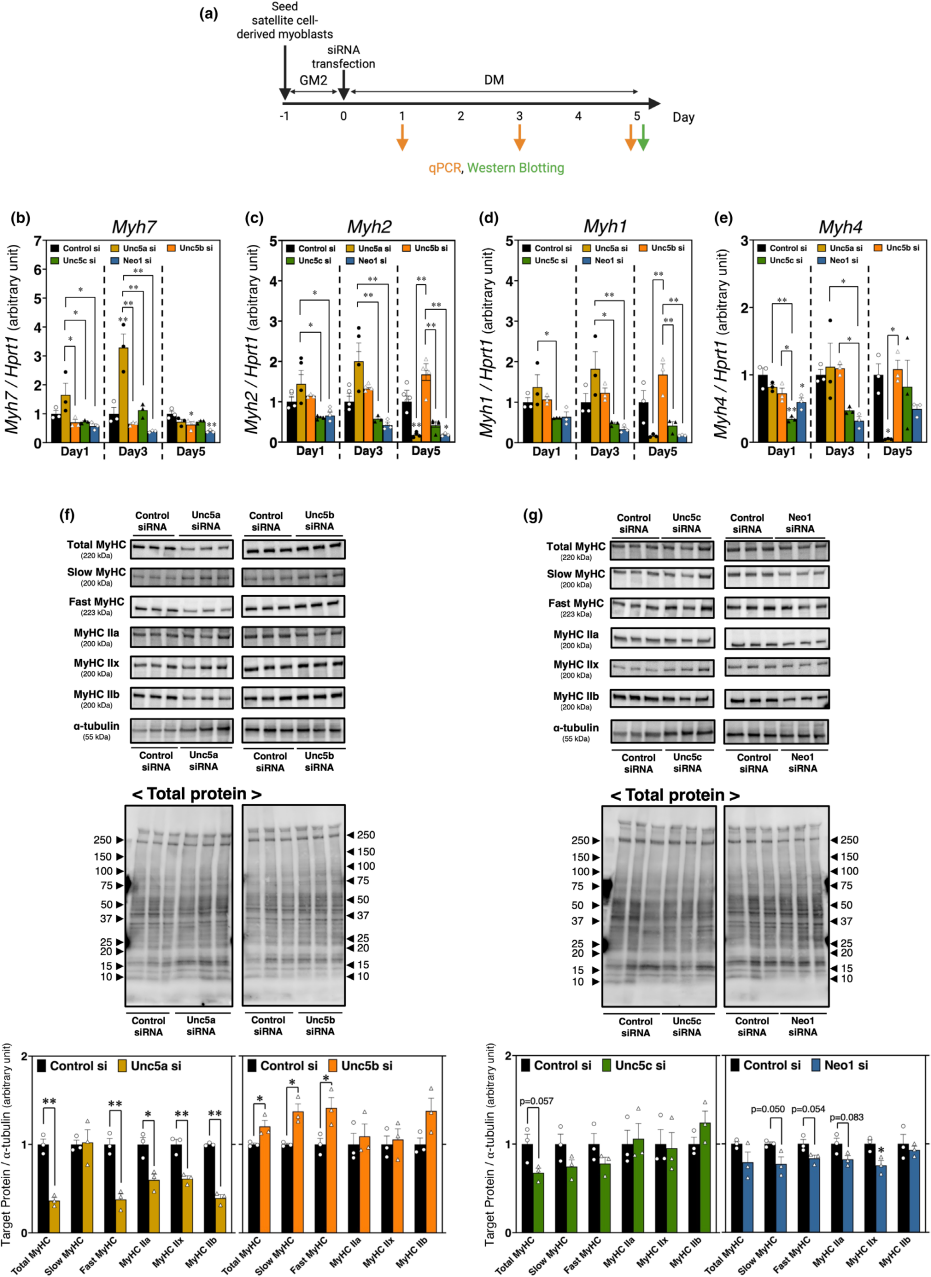
The expression and synthesis patterns of myosin heavy chain isoforms suppressing UNC5A, UNC5B, UNC5C, or neogenin expression in satellite cell‐derived myoblast cultures. (a) Time course of UNC5A, UNC5B, UNC5C, or neogenin knockdown. (b–e) qPCR analysis of myosin heavy chain (MyHC) isoform ([b]*Myh7*, [c]*Myh2*, [d]*Myh1*, and [e]*Myh4*) mRNA expression in myoblasts with UNC5A, UNC5B, UNC5C, or neogenin knockdown on 1, 3, and 5 days after induction of myogenic differentiation; *HPRT1* expression was used as an internal control. A unique color represents each knockdown group (bars are color‐coded to correspond to their respective groups [UNC5A; yellow ochre, UNC5B; orange, UNC5C; green, and neogenin; blue]). The bars depict arbitrary units (means ± SEMs of three independent cultures) relative to control siRNA‐treated myoblast cultures (the black bar). Statistical comparisons were conducted within each time point. Significant differences from the control siRNA group mean at *p* < 0.05 and *p* < 0.01 are indicated by single and double asterisks, respectively (one‐way ANOVA followed by Tukey–Kramer post‐hoc test; *n* = 3). (f and g) Western Blotting analysis of MyHC isoform protein expression in myoblasts with knockdown of UNC5A, UNC5B, UNC5C, or neogenin after 5 days of myogenic differentiation, with α‐tubulin and total protein used as loading controls. The graph presents the quantified band intensities, normalized to α‐tubulin. The uncropped images of Western Blotting analysis are shown in Figure S2 (UNC5A or UNC5B knockdown: [S2A]Total MyHC, [S2B]Slow MyHC, [S2C]Fast MyHC, [S2D]MyHC type IIa, [S2E]MyHC type IIx, [S2F]MyHC type IIb, and [S2G]α‐tubulin; UNC5C or Neogenin knockdown: [S2H]Total MyHC, [S2I]Slow MyHC, [S2J]Fast MyHC, [S2K]MyHC type IIa, [S2L]MyHC type IIx, [S2M]MyHC type IIb, and [S2N]α‐tubulin). Data are presented as the mean ± SEM. The bars depict arbitrary units relative to control siRNA‐treated myoblast cultures (the black bar). Significant differences from the control siRNA group mean at *p* < 0.05 and *p* < 0.01 are indicated by single and double asterisks, respectively (Student's *t*‐test; *n* = 3).

Satellite cell‐derived myoblasts were established from the back, buttock, and upper hindlimb muscles of adult C57BL/6 mice and cultured as previously described (Maeno et al., 2023; Ojima et al., 2004; Suzuki et al., 2021). Briefly, myoblasts were plated in 12‐ (5.0 × 10^4^ cells/well) or 24‐well plates (2.5 × 10^4^ cells/well) coated with collagen type I (cat. no. 637‐00653, Cellmatrix Type I‐A; Nitta Gelatin Bland, Kurabo, Osaka, Japan). Cells were maintained in Growth Medium 2 [GM2; Ham's F10 Nutrient Mix (cat. no. 11550043, Gibco) containing 20% FBS, 0.5% gentamicin, 1% AA‐mix, and 2.5 ng/mL FGF2] for 1 day, after which the medium was replaced with siRNA‐containing DM (Figures 2a, 3a, 6a and 7a). All cultures were maintained in a humidified atmosphere with 5% CO_2_ at 37°C.

### 2.4 RNA interference and recombinant protein addition

We performed a loss‐of‐function (knockdown) experiment using siRNA targeting UNC5A, UNC5B, UNC5C, and neogenin as previously described (Maeno et al., 2023; Suzuki et al., 2021). Briefly, proliferating cells were transfected with 40 nM mouse *Unc5a*‐, *Unc5b*‐, *Unc5c*‐, and *Neo1*‐specific siRNAs using ScreenFect™ siRNA Transfection Reagent (cat. no. 295‐75,003, FUJIFILM Wako Pure Chemical Industries, Osaka, Japan) according to the manufacturer's recommendations; the culture medium was switched to DM at this time. The medium was replaced with DM supplemented with 50 ng/mL recombinant mouse netrin‐1 (cat. no. 1109‐N1, R&D Systems) or recombinant mouse netrin‐4 (cat. no. 1132‐N4, R&D Systems) 1 day after UNC5A knockdown (Figure 6a). AllStar negative control siRNA (cat. no. 1027281, Qiagen, Hilden, Germany) was used for control samples. Stealth RNAi siRNAs were designed using BLOCK‐iT RNAi Designer (Invitrogen, Thermo Fisher Scientific, Waltham, MA, USA [https://rnaidesigner.thermofisher.com/rnaiexpress/]). Transfected cells were maintained in DM until analysis. The siRNA sequences are listed in Table 1.

**TABLE 1:** List of siRNA sequences.

siRNA	Sense (5′ to 3′)	Antisense (5′ to 3′)
Unc5a siRNA (Stealth_661)	GGCCUACAUCCGGAUUGCCUAUUUG	CAAAUAGGCAAUCCGGAUGUAGGCC
Unc5b siRNA (Stealth_636)	GCCACGCAGAUCUACUUCAAGUGUA	UACACUUGAAGUAGAUCUGCGUGGC
Unc5c siRNA (Stealth_2778)	CAACUGGCGUAAUCCUGGAUCUUUG	CAAAGAUCCAGGAUUACGCCAGUUG
Neo1 siRNA (Stealth_3011)	CAGAAGAUUACAGACUCCCGCUACU	AGUAGCGGGAGUCUGUAAUCUUCUG

### 2.5 Reverse transcription‐quantitative polymerase chain reaction

Total RNA was harvested from cells using ISOGEN II reagent (cat. no. 311‐07361, Nippon Gene, Tokyo, Japan), following the manufacturer's protocol. Complementary DNA (cDNA) was synthesized from the extracted RNA using a ReverTra Ace qPCR RT Kit (cat. no. FSQ‐101, TOYOBO, Osaka, Japan).

#### 2.5.1 qPCR

Quantitative analysis of mRNA expression (qPCR) was performed using THUNDERBIRD NEXTSYBR qPCR Mix (cat. no. QPX‐201, TOYOBO). Reactions were performed on a LightCycler 96 instrument (cat. no. 05815916001, Roche Diagnostics, Mannheim, Germany). Annealing and extension were uniformly performed at 60°C for all assays, according to the manufacturer's protocol. Quantitative analysis was performed using LightCycler 96 software (version 1.1, Roche Diagnostics). mRNA levels were normalized to those of *HPRT1*. The primer sequences are listed in Table 2.

**TABLE 2:** List of primer sets for qPCR.

Primer name	Primer sequence (5′ to 3′) forward	Primer sequence (5′ to 3′) reverse
*Myh7* (MyHC I)	CAAGGTCAATACTCTGACCAAGG	CCATGCGCACCTTCTTCT
*Myh2* (MyHC IIa)	AGAGGGTTCTTGGCGAGAGT	AAGGCGCGGATGTTGTACT
*Myh1* (MyHC IIx)	TCTGCAGACGGAGTCAGGT	TTGAGTGAATGCCTGTTTGC
*Myh4* (MyHC IIb)	TGATGCAGGAGAAAAATGACC	ATCAGCTGGTCGCACCTTT
*Myod1* (MyoD)	AGCACTACAGTGGCGACTCA	GGCCGCTGTAATCCATCA
*Myog* (Myogenin)	CCTTGCTCAGCTCCCTCA	TGGGAGTTGCATTCACTGG
*Myf6* (Myf6)	AGGGGCCTCGTGATAACTG	GGAAGAAAGGCGCTGAAGA
*Mymk* (Myomaker)	CCACCCTCATCATTGCTGTA	GTAGATGCTCTTGTCGGGGTA
*Mymx* (Myomixer)	CCACTGGCCGGTTAGAACT	CATCGGGAGCAATGGAAC
*Unc5a* (UNC5A)	GGGCAGAATGTCCAGAAAAC	GCAGGCTGACCACTTACTCC
*Unc5b* (UNC5B)	TTCCAGCTGCACACAACG	GCAGAGCAGAGAGCATCCA
*Unc5c* (UNC5C)	GCACAGACCCCAGAATGAAT	CCCACGTAGAGAGCCACATC
*Neo1* (Neogenin)	GGGGAATGAGACCAAAAATG	GGATGGGCACTAATCACAGG
*Mafa* (MafA)	AGGCCACCACGTGCGCTTGG	GCTGCTGCACCCGCTTGAAG
*Mafb* (MafB)	CATCACCATCATCACCAAGC	AGAAGCGGTCCTCCACACTA
*Maf* (Maf)	GCAATGAACAATTCCGACCT	CCGGTTCCTTTTTCACTTCA
*Six1* (Six1)	CTTTAAGGAGAAGTCTCGGG	TTCCAGAGGAGAGAGTTGAT
*Six4* (Six4)	CCACGGTTTTTCCCTGACCC	GGTTGCATAGTTAGTGTTGCTGA
*Smarcd3* (Baf60c)	AGGCTTACATGGACCTCCTAG	CATCAGAGTCTTCCGCATCAG
*Sox6* (Sox6)	AATGCACAACAAACCTCACTCT	AGGTAGACGTATTTCGGAAGGA
*Tbx15* (Tbx15)	TGTTCGCACACTGACCTTTG	CCAGTGCTGGAGGTGGTT
*Hprt1* (HPRT)	CCTCCTCAGACCGCTTTTT	AACCTGGTTCATCATCGCTAA

#### 2.5.2 semi‐qPCR

Semi‐reverse transcription quantitative PCR (Semi‐qPCR) was performed using a KOD FX DNA polymerase mixture (cat. no. KFX‐101, TOYOBO), as previously described (Suzuki et al., 2021). Amplification was performed with initial denaturation at 94°C for 2 min, followed by 28–37 cycles of 10‐s denaturation at 98°C, 30‐s annealing at 61°C–66°C, and 60‐s extension at 68°C. The PCR products were separated using 2.0% agarose gel electrophoresis and visualized using GelRed (cat. no. 518‐24031, Biotium, Fremont, CA, USA). mRNA levels were normalized to those of *HPRT1*. The primer sets are listed in Table 3.

**TABLE 3:** List of primer sets for Semi‐qPCR.

Primer name	Primer sequence (5′ to 3′) forward	Primer sequence (5′ to 3′) reverse	Amplicon (bp)	Cycle
*Unc5a*	GAGTCGCCCTCTCATCTCTACTACTG	GTTAAAGTTGATGTTGAAGCTCTGTCCATC	519	33
*Unc5b*	AACTAAGTAGACAGCTGTATACCCACTCTT	CACAATCAACTTGTCTCTACTCGATTCTCA	552	29
*Unc5c*	CTATTCAGAGATATGTGCTGGTGTAAGTCC	GAGCTTAAGTGTCAGCTTTTTAGAAACAGG	563	34
*Unc5d*	TCAAGAAGGGAGATAGACCTCAAACTAGTT	CTTCCTTGGAGACATTTATTCACTCACTCA	592	37
*DCC*	GTATCTACGGCTTGAAACCAGCTGAATATA	CACATAGTTCCTCGAAAGTACTTCCAGAAA	533	37
*Neo1*	TGAATTGTGAAGTTAATGCAGATTTGGTCC	TGTCCATGGATTCGTGAGCATATATGTTAG	561	30
*Hprt1*	CCGAGGATTTGGAAAAAGTGTTTATTCCTC	CTTTTCCAGTTTCACTAATGACACAAACGT	555	28

### 2.6 Western Blotting using enhanced chemiluminescence (ECL)

Whole‐cell lysates from cell cultures were subjected to 4%–15% precast polyacrylamide gel electrophoresis (cat. no. 4561085, Bio‐Rad Laboratories, Hercules, CA, USA) under reducing conditions and subsequently transferred to polyvinylidene fluoride membranes (cat. no. 033‐22,453, FUJIFILM Wako). Proteins were visualized using No‐Stain Protein Labeling Reagent (cat. no. A44449, Thermo Fisher Scientific) according to the manufacturer's instructions, and the membrane was imaged using a FUSION SOLO 7S Edge (M&S Instruments, Osaka, Japan). Membranes were blocked with 5% nonfat skimmed dry milk in 0.1% T‐TBS for 40 min at 25°C and then incubated overnight at 4°C with antigen affinity‐purified primary antibodies. Primary antibodies were diluted in Can Get Signal Solution 1 (cat. no. NKB‐201, TOYOBO). After washing with 0.1% T‐TBS, membranes were treated with secondary antibodies diluted in Can Get Signal solution 2 (cat. no. NKB‐301, TOYOBO) for 1 h at 25°C. The antibodies used are listed in Table 4. Immunoreactive signals were visualized using Enhanced chemiluminescence (ECL) or ECL Select Western Blotting Detection Reagent (cat. no. RPN2106 or RPN2235, Cytiva), and bands were captured using LuminoGraph I (ATTO, Tokyo, Japan).

**TABLE 4:** List of antibodies for western blotting and Immunocytochemistry (ICC).

Antibodies	Species	Antibody reference	Supplier	Dilution *for ICC
UNC5A	Rabbit	20,239‐1‐AP	Proteintech	1:500
UNC5B	Goat	AF1006	R&D Systems	1:500
UNC5C	Goat	AF1005	R&D Systems	1:1000
Neogenin	Goat	AF1079	R&D Systems	1:500
Total MyHC	Mouse	MAB4470	R&D Systems	1:500 *1:50
Slow MyHC	Mouse	M8421	Sigma‐Aldrich	1:500 *1:50
Fast MyHC	Mouse	M4276	Sigma‐Aldrich	1:500 *1:50
MyHC IIa	Rat	Sawano et al. (2016)	‐	1 μg/mL
MyHC IIx	Rat	Sawano et al. (2016)	‐	1 μg/mL
MyHC IIb	Rat	Sawano et al. (2016)	‐	1 μg/mL
α‐tubulin	Mouse	M175‐3	Medical & Biological Laboratories	1:2000
Horseradish peroxidase (HRP)‐conjugated anti‐mouse IgG	Rabbit	315‐036‐003	Jackson ImmunoResearch Laboratories Inc.	1:5000
HRP‐conjugated anti‐goat IgG	Rabbit	305‐036‐003	Jackson ImmunoResearch Laboratories Inc.	1:5000
HRP‐conjugated anti‐rat IgG	Goat	112‐036‐003	Jackson ImmunoResearch Laboratories Inc.	1:5000
HRP‐conjugated anti‐rabbit IgG	Goat	111‐036‐003	Jackson ImmunoResearch Laboratories Inc.	1:5000
HRP‐conjugated anti‐sheep IgG	Donkey	713‐036‐147	Jackson ImmunoResearch Laboratories Inc.	1:5000
*Anti‐mouse IgG antibody Texas Red conjugated	*Goat	*610‐1919	*Rockland Immunochemicals	*1:250

### 2.7 Immunocytochemistry

Cells were fixed with 4% paraformaldehyde in PBS for 10 min at 25°C. Fixed cells were permeabilized with 0.2% Triton X‐100 (cat. no. X100‐100ML, Sigma‐Aldrich, St. Louis, MO, USA) for 15 min and blocked with 3% bovine serum albumin in 0.1% polyethylene sorbitan monolaurate (Tween 20)‐PBS for 40 min at 25°C. After blocking, cells were incubated with primary monoclonal antibodies diluted in blocking solution overnight at 4°C. Subsequently, cells were probed with secondary antibodies diluted in blocking solution for 1 h at 25°C. As well as Western Blotting, the antibodies used are listed in Table 4. Mounted samples were prepared using ProLong Diamond Antifade Mountant with 4′,6‐diamidino‐2‐phenylindole (DAPI; cat. no. P36962, Thermo Fisher Scientific) and visualized using a DMI6000B confocal microscope (Leica, Wetzlar, Germany). We analyzed the myotube fusion index of MyHC isoform‐positive cells, myotube area, and myotube diameter from 10 images randomly captured per well using Myotube Analyzer (Noë et al., 2022). Briefly, the images stained with DAPI, fast MyHC, and slow MyHC were thresholded to eliminate background noise, processed into binary masks, and the channels were overlaid to quantify the number of nuclei co‐localizing with fast MyHC‐ or slow MyHC‐positive myotubes. The myotube fusion index was calculated as the percentage of nuclei overlapping with fast or slow MyHC‐positive myotubes relative to the total number of nuclei within the field of view.

### 2.8 Statistical analysis

Data are presented as the mean ± standard error of means (SEM).

**FIGURE 4:**
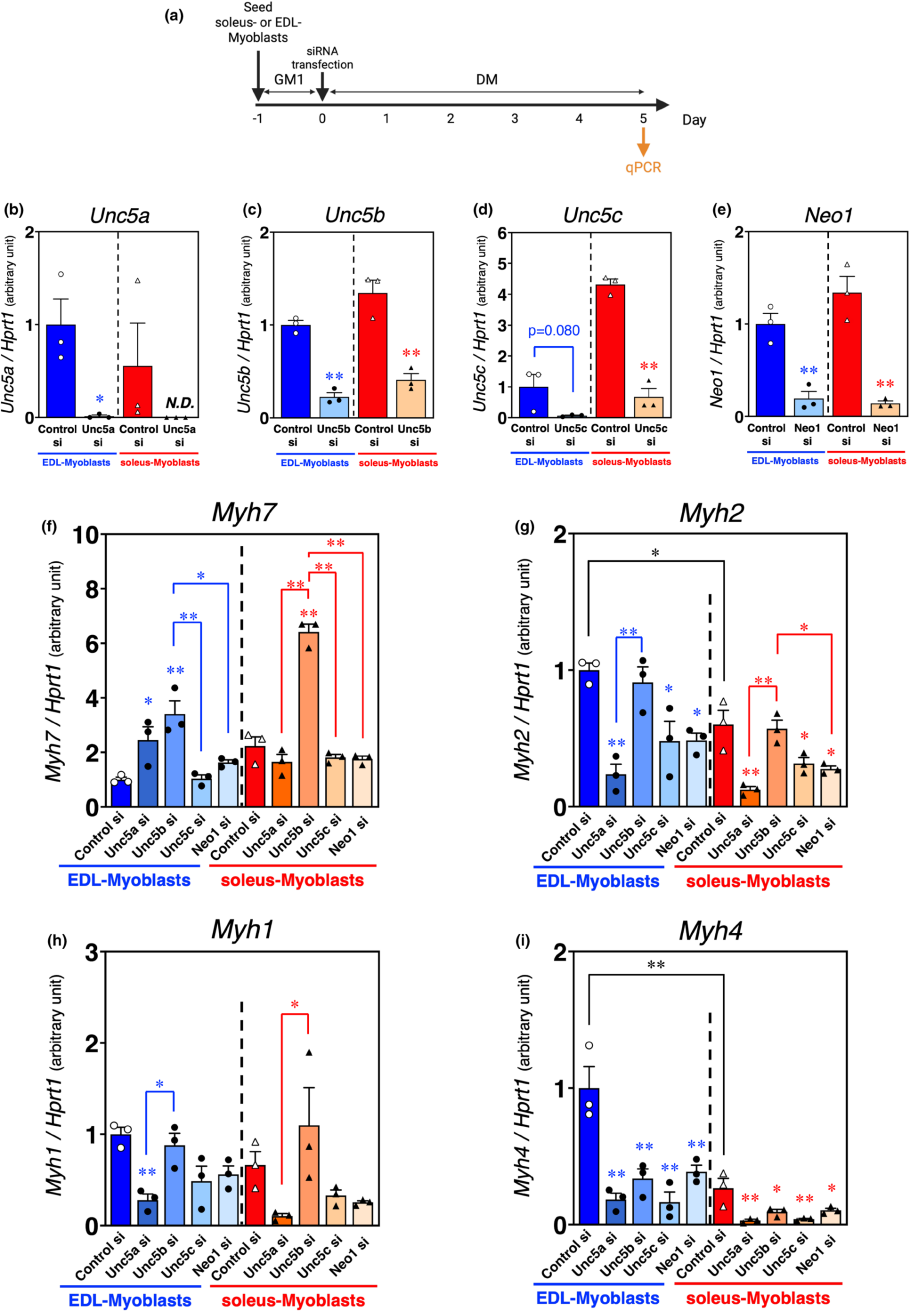
The mRNA expressions of myosin heavy chain isoforms suppressing UNC5A, UNC5B, UNC5C, or neogenin expression in both soleus and EDL myoblasts. (a) Time course of UNC5A, UNC5B, UNC5C, or neogenin depletion through RNA interference in EDL and soleus myoblasts. (b–i) qPCR analysis of netrin receptors ([b]*Unc5a*, [c]*Unc5b*, [d]*Unc5c*, and [e]*Neo1*) and myosin heavy chain isoforms ([f]*Myh7*, [g]*Myh2*, [h]*Myh1* and [i]*Myh4*) in EDL and soleus myoblasts following knockdown of UNC5A, UNC5B, UNC5C, or neogenin on day 5 of myogenic differentiation; *HPRT1* expression was used as an internal control. The bars depict arbitrary units (means ± SEMs of three independent cultures) relative to EDL myoblasts treated with control siRNA. Statistical comparisons were conducted within each myoblast type (soleus and EDL), and asterisks indicated by red and blue represent soleus and EDL myoblasts, respectively. Significant differences at *p* < 0.05 and *p* < 0.01 are indicated by single and double asterisks, respectively ([b–e]: Student's *t*‐test; *n* = 3; [f–i]: One‐way ANOVA followed by Tukey–Kramer post‐hoc test; *n* = 3).

Differences between two groups (Figures 2b–e, 3f,g, 4b–e) were assessed using Student's *t*‐test in Microsoft Excel for Mac (Version 16.94, Microsoft Corporation, Redmond, WA, USA). Statistical significance was defined as *p* < 0.05 or *p* < 0.01, represented by * and **, respectively.

**FIGURE 5:**
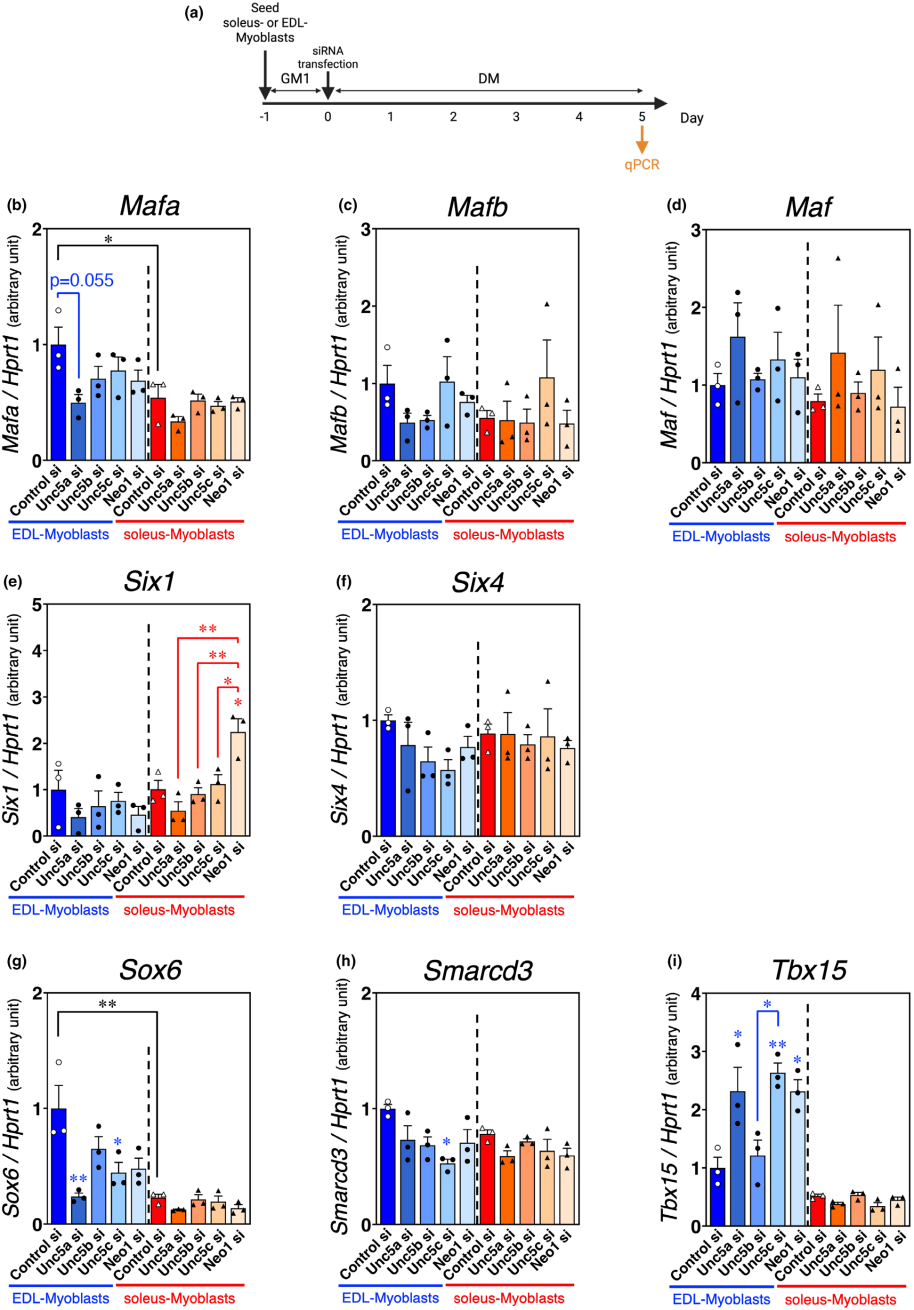
The mRNA expression patterns of transcription factors associated with fast‐twitch myofiber formation following netrin receptor suppression in both soleus and EDL myoblasts. (a) Time course of UNC5A, UNC5B, UNC5C, or neogenin depletion through RNA interference in EDL and soleus myoblasts, as well as performed in Figure 4. (b–i) qPCR analysis of transcription factors associated with fast‐twitch myofiber formation ([a]*Mafa*, [c]*Mafb*, [d]*Maf*, [e]*Six1*, [f]*Six4*, [g]*Sox6*, [h]*Smarcd3*, and [i]*Tbx15*) in EDL and soleus myoblasts following knockdown of UNC5A, UNC5B, UNC5C, or neogenin on Day 5 of myogenic differentiation; *HPRT1* expression was used as an internal control. The bars depict arbitrary units (means ± SEMs of three independent cultures) relative to EDL myoblasts treated with control siRNA. Statistical comparisons were conducted within each myoblast type (soleus and EDL), and asterisks indicated by red and blue represent soleus and EDL myoblasts, respectively. Significant differences at *p* < 0.05 and *p* < 0.01 are indicated by single and double asterisks, respectively (one‐way ANOVA followed by Tukey–Kramer post‐hoc test; *n* = 3).

**FIGURE 6:**
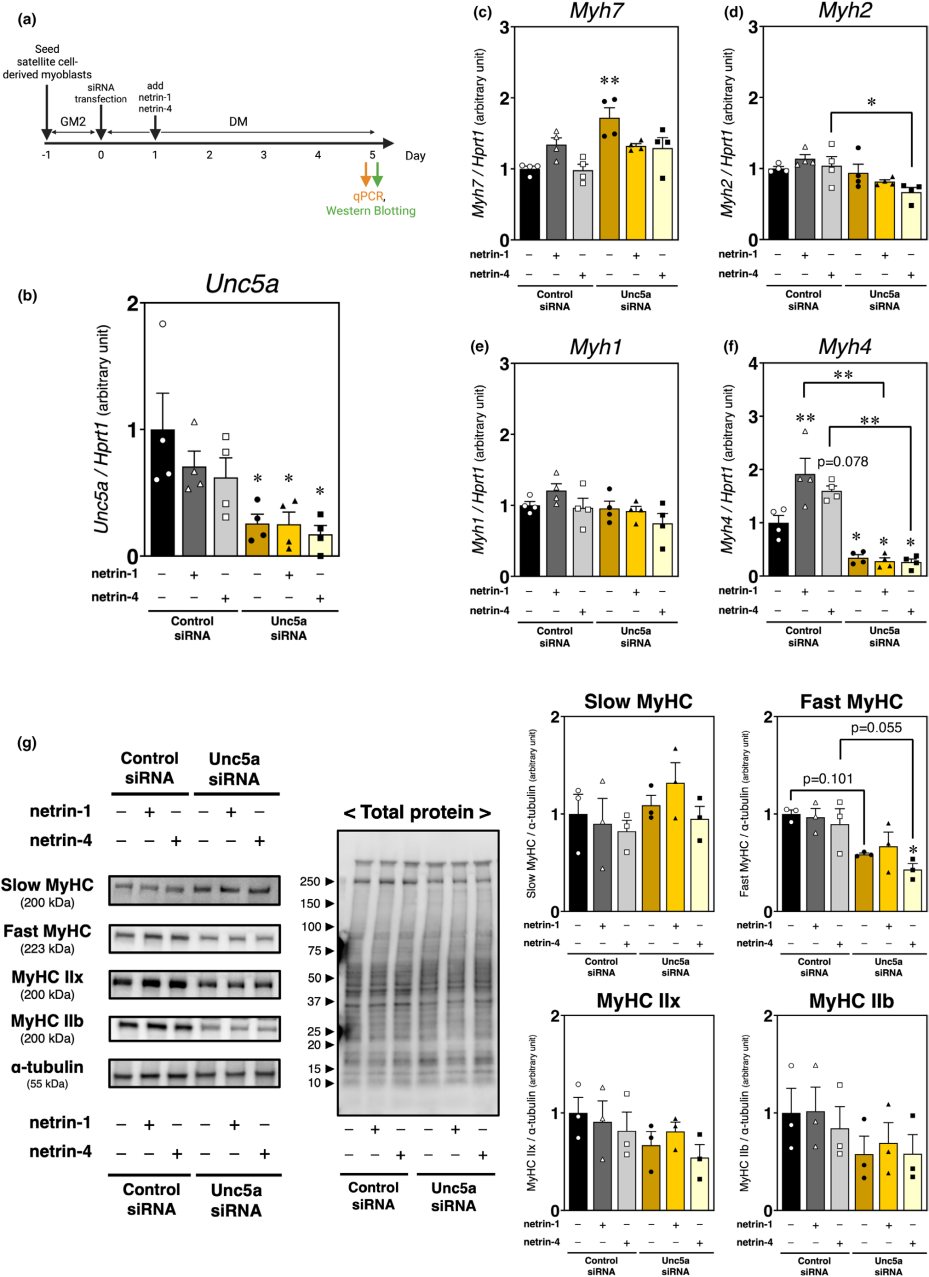
A combination experiment involving UNC5A suppression and the addition of recombinant netrin‐1 or netrin‐4. (a) Time course of recombinant netrin‐1 or netrin‐4 treatment following UNC5A siRNA treatment in myoblasts during myogenic differentiation. (b–f) qPCR analysis of *Unc5a* and myosin heavy chain (MyHC) isoform mRNA expression in UNC5A‐knockdown myoblasts supplemented with recombinant netrin‐1 or netrin‐4 after 5 days of myogenic differentiation; *HPRT1* expression was used as an internal control. (g) Western Blotting analysis of MyHC isoform protein expression in UNC5A‐knockdown myoblasts supplemented with recombinant netrin‐1 or netrin‐4, with α‐tubulin and total protein used as loading controls. Band intensities were quantified and normalized to α‐tubulin, as shown in the graph. The uncropped images of Western Blotting analysis are provided in Figure S3 ([S3A]Slow MyHC, [S3B]Fast MyHC, [S3C]MyHC type IIx, [S3D]MyHC type IIb, and [S3E]α‐tubulin). The bars depict arbitrary units (means ± SEMs of three or four independent cultures) relative to control siRNA‐transfected myoblasts without additional recombinant netrins (the black bar). Significant differences from the control siRNA myoblasts without additional netrins are indicated by single and double asterisks for *p* < 0.05 and *p* < 0.01, respectively (one‐way ANOVA followed by Tukey–Kramer post‐hoc test; [b–f]: *n* = 4, [g]: *n* = 3).

**FIGURE 7:**
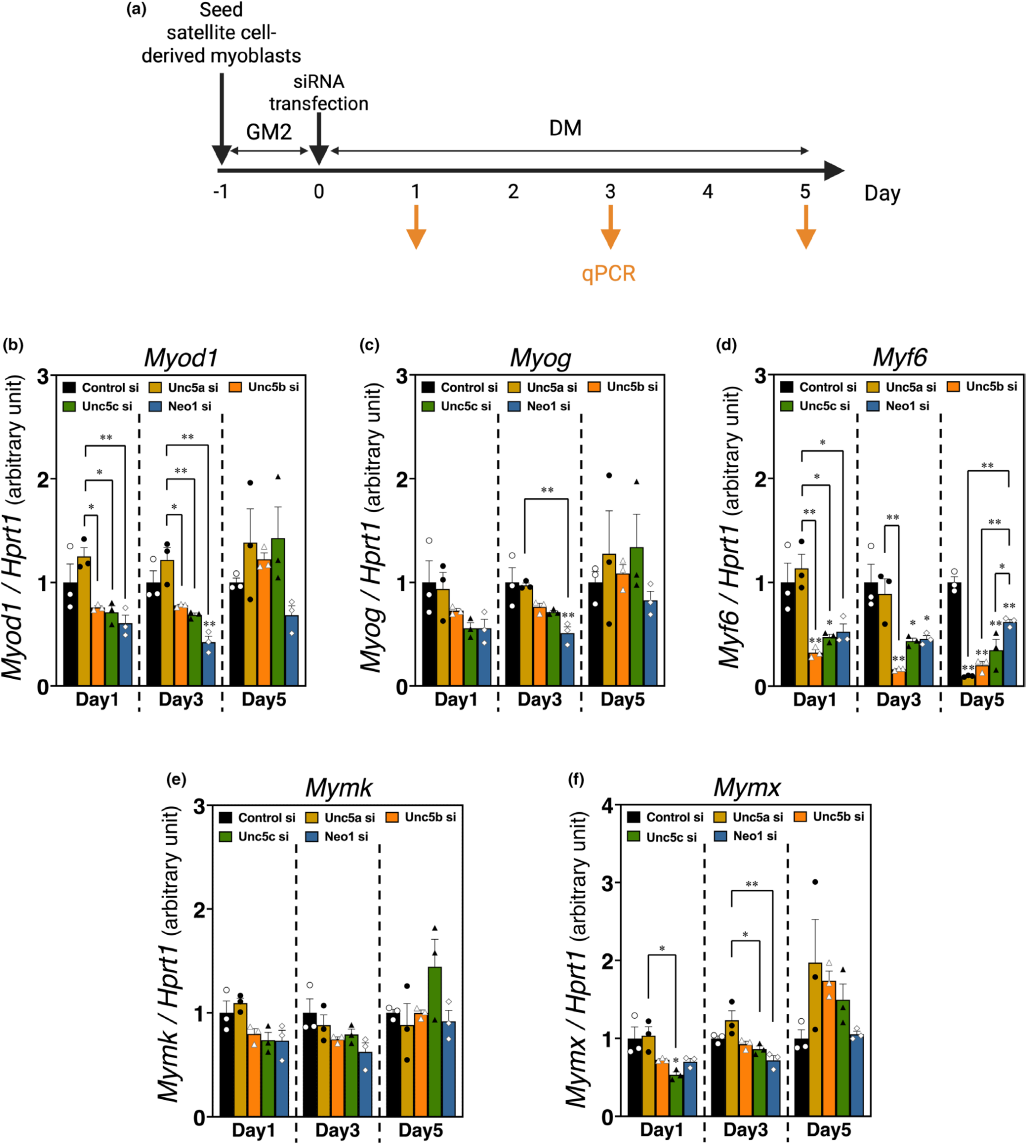
Silencing of UNC5A, UNC5B, UNC5C, and neogenin affected the mRNA expression of myogenic transcription factors and myoblast fusion factors during myogenic differentiation. (a) Time course of UNC5A, UNC5B, UNC5C, or neogenin knockdown, as well as performed in Figures 2 and 3. (b–f) qPCR analysis of mRNA expression associated with myogenic transcription factors ([b]*Myod1*, [c]*Myog*, and [d]*Myf6*) and myoblast fusion factors ([e]*Mymk* and [f]*Mymx*) in myoblasts with knockdown of UNC5A, UNC5B, UNC5C, or neogenin 1, 3, and 5 days post‐differentiation; *HPRT1* expression was used as an internal control. Each group is represented by a unique color (UNC5A; yellow ochre, UNC5B; orange, UNC5C; green, and neogenin; blue). The bars depict arbitrary units (means ± SEMs of three independent cultures) relative to control siRNA‐treated myoblast cultures (the black bar). Statistical comparisons were conducted within each time point. Significant differences from the control siRNA group mean at *p* < 0.05 and *p* < 0.01 are indicated by single and double asterisks, respectively (one‐way ANOVA followed by Tukey–Kramer post‐hoc test; *n* = 3).

For comparisons among multiple groups (Figures 1b,d–h, 2f,g, 3b,c–e, 4f–i, 5b–i, 6b–g, 7b–f), statistical analyses were performed using one‐way analysis of variance (ANOVA) followed by Tukey–Kramer post‐hoc tests using EZR (Saitama Medical Center, Jichi Medical University, Saitama, Japan) which is a graphical user interface in R software (version 3.6.2; The R Foundation for Statistical Computing, Vienna, Austria) (Kanda, 2013). Statistical significance was set at a *p*‐value of less than 0.05 or 0.01, indicated by * or **, respectively.

## 3 RESULTS

### 3.1 UNC5A, UNC5B, UNC5C, and neogenin were synthesized in myoblasts

We examined the expression patterns of UNC5A, UNC5B, UNC5C, UNC5D, neogenin, and DCC in soleus and EDL myoblasts during myogenic differentiation using semi‐qPCR, qPCR, and Western Blotting (Figure 1a). First, we confirmed that *Myh7* (MyHC type I mRNA) expression was significantly higher in soleus than in EDL myoblasts on Day 5 post‐differentiation (*p* = 0.0005); *Myh4* (MyHC type IIb mRNA) expression was not statistically higher in EDL myoblasts (Figure 1b). However, *Myh7* and *Myh4* expression levels were significantly upregulated at Day 5 during the myogenic differentiation process, following a time‐dependent pattern (*Myh7*: *p* = 0.0006 vs. Day 0; *p* = 0.0004 vs. Day 1; *p* = 0.0247 vs. Day 3; *Myh4*: *p* = 0.0174 vs. Day 0; *p* = 0.0201 vs. Day 1). These data are consistent with our previous finding (Maeno et al., 2023), indicating that our culture conditions were suitable for supporting the formation of myotubes that retain myofiber‐type specificity based on their myoblast origin. *Unc5d* and *Dcc* were scarcely expressed during myogenic differentiation, whereas *Unc5a*, *Unc5b*, *Unc5c*, and *Neo1* were constitutively expressed regardless of the myoblast origin (Figure 1c).

In a time‐dependent manner during the myogenic differentiation process, *Unc5a* expression increased in both soleus and EDL myoblasts (soleus: *p* = 0.0350 [Day1 vs. Day5]; EDL: *p* = 0.0561 [Day1 vs. Day5]) (Figure 1d), whereas *Unc5b* expression decreased compared to Day0 (soleus: *p* = 0.0433 vs. Day1, *p* = 0.0040 vs. Day3, *p* = 0.0424 vs. Day5; EDL: *p* = 0.0003 vs. Day1, *p* = 0.0001 vs. Day3, *p* = 0.0002 vs. Day5) (Figure 1e). *Unc5c* expression decreased specifically in soleus myoblasts (*p* = 0.0003 [Day0 vs. Day3], *p* = 0.0009 [Day0 vs. Day5], *p* = 0.0122 [Day1 vs. Day3], *p* = 0.0343 [Day1 vs. Day3]) (Figure 1f), and *Neo1* expression was downregulated in EDL myoblasts compared to Day0 (*p* = 0.025 vs. Day1, *p* = 0.036 vs. Day5) (Figure 1g).

When focusing on the expression profiles based on myoblast origins, *Unc5a* and *Unc5b* expression levels did not differ between soleus myoblasts and EDL myoblast cultures (Figure 1d,e). *Unc5c* expression was higher in soleus myoblasts during myogenic differentiation (*p* = 0.000005 [Day0], *p* = 0.000044 [Day1], *p* = 0.026616 [Day3], *p* = 0.058710 [Day5]) (Figure 1f), and *Neo1* expression was higher in soleus than in EDL myoblasts 5 days after the induction of differentiation (*p* = 0.039) (Figure 1c).

Although the protein synthesis patterns of UNC5A were not consistent with their mRNA expression profiles in a time‐dependent manner, the bands observed in EDL myoblasts on Day1 and Day2 appeared to be upregulated compared to those on Day0 (Figure 1h). UNC5C synthesis level decreased specifically in EDL myoblasts compared to Day0 (*p* = 0.019 vs. Day1, *p* = 0.022 vs. Day2, *p* = 0.021 vs. Day3, *p* = 0.001 vs. Day4, *p* = 0.003 vs. Day5). The band patterns of UNC5A, UNC5B, UNC5C, and neogenin were not quantitatively significant; however, they appeared to indicate higher protein levels in EDL myoblasts.

These data suggest that UNC5A, UNC5B, UNC5C, and neogenin, synthesized in myoblasts, may participate in the autonomous regulation of myofiber type.

### 3.2 Myotube formation is inhibited by UNC5A‐, UNC5B‐, UNC5C‐, and neogenin knockdown; UNC5A knockdown significantly impairs fast‐twitch myotube formation

We performed knockdown experiments using siRNAs in satellite cell‐derived myoblasts to investigate the roles of UNC5A, UNC5B, UNC5C, and neogenin in the formation of fast‐ and slow‐twitch myotubes (Figures 2a and 3a). siRNA transfection at the onset of myogenic differentiation significantly and stably inhibited the expression of each netrin receptor at 1 day (*Unc5a*: *p* = 0.006; *Unc5b*: *p* = 0.005; *Unc5c*: *p* = 0.012; *Neo1*: *p* = 0.003), 3 days (*Unc5a*: *p* = 0.096; *Unc5b*: *p* = 0.021; *Unc5c*: *p* = 0.0002; *Neo1*: *p* = 0.017), and 5 days (*Unc5a*: *p* = 0.001; *Unc5b*: *p* = 0.008; *Unc5c*: *p* = 0.012; *Neo1*: *p* = 0.003) post‐differentiation (Figure 2b–e). After 5 days, the knockdown of any receptor resulted in a reduced fusion index, myotube area, and myotube diameter in fast MyHC‐positive myotubes (fusion index: *p* = 0.000003 [Control si vs. Unc5a si], *p* = 0.0001 [Control si vs. Unc5b si], *p* = 0.0003 [Control si vs. Unc5c si], *p* = 0.00002 [Control si vs. Neo1 si]; myotube area: *p* = 0.000003 [Control si vs. Unc5a si], *p* = 0.0001 [Control si vs. Unc5b si], *p* = 0.000007 [Control si vs. Unc5c si], *p* = 0.000003 [Control si vs. Neo1 si], *p* = 0.023 [Unc5a si vs. Unc5b si], *p* = 0.020 [Unc5b si vs. Neo1 si]; myotube diameter: *p* = 0.00002 [Control si vs. Unc5a si], *p* = 0.00001 [Control si vs. Unc5b si], *p* = 0.00002 [Control si vs. Unc5c si], *p* = 0.00002 [Control si vs. Neo1 si]) (Figure 2f). Notably, the fusion index of fast MyHC‐positive myotubes was significantly lower in UNC5A‐knockdown myoblasts than in UNC5B‐ or UNC5C‐knockdown myoblasts (*p* = 0.024 [Unc5a si vs. Unc5b si], *p* = 0.005 [Unc5a si vs. Unc5c si]). In slow MyHC‐positive myotubes, the fusion index and diameter decreased following the knockdown of any receptor (fusion index: *p* = 0.0010 [Control si vs. Unc5a si], *p* = 0.0222 [Control si vs. Unc5b si], *p* = 0.0237 [Control si vs. Unc5c si], *p* = 0.0005 [Control si vs. Neo1 si]; myotube diameter: *p* = 0.000004 [Control si vs. Unc5a si], *p* = 0.000005 [Control si vs. Unc5b si], *p* = 0.000008 [Control si vs. Unc5c si], *p* = 0.000019 [Control si vs. Neo1 si]); however, the myotube area was not significantly reduced exclusively in UNC5B‐knockdown myoblasts (myotube area: *p* = 0.0051 [Control si vs. Unc5a si], *p* = 0.1136 [Control si vs. Unc5b si], *p* = 0.0006 [Control si vs. Unc5c si], *p* = 0.0002 [Control si vs. Neo1 si], *p* = 0.0356 [Unc5b si vs. Unc5c si], *p* = 0.0079 [Unc5b si vs. Neo1 si]) (Figure 2g). Considering these morphological data of both fast‐ and slow‐twitch myotubes formation, each netrin receptor may not be specifically involved in the myofiber type regulation.

We further clarified the impact of netrin receptor knockdown on the myofiber types of myotubes based on the mRNA and protein levels of MyHC isoforms (Figure 3). UNC5A knockdown resulted in either no change or a significant increase in the mRNA levels of all MyHC isoforms compared with the control siRNA group by post‐differentiation 3 days (Figure 3b–e). However, when focusing on other netrin receptors, UNC5A knockdown induced higher expression levels of *Myh2* (MyHC type IIa mRNA) on Days 1 and 3 post‐differentiation, corresponding to the middle stage of myogenic differentiation (Day 1: *p* = 0.0309 [Unc5a si vs. Unc5c si], *p* = 0.0438 [Unc5a si vs. Neo1 si]; Day 3: *p* = 0.0062 [Unc5a si vs. Unc5c si], *p* = 0.0030 [Unc5a si vs. Neo1 si]) (Figure 3c). Similar phenotypes were also observed for *Myh1* (MyHC type IIx mRNA) (Day 1: *p* = 0.0443 [Unc5a si vs. Unc5c si]; Day 3: *p* = 0.0105 [Unc5a si vs. Unc5c si], *p* = 0.0054 [Unc5a si vs. Neo1 si]) (Figure 3d). On the contrary, on 5 days, *Myh2* (*p* = 0.009), *Myh1* (*p* = 0.054), and *Myh4* levels (*p* = 0.045) were remarkably reduced in UNC5A knockdown myoblasts compared with the control group (Figure 3c–e). Consistent with the mRNA analysis, UNC5A knockdown reduced the fast‐type MyHC, which recognized all type II isoforms, protein synthesis (*p* = 0.0025); reductions included MyHC type IIa (*p* = 0.0178), IIx (*p* = 0.0044), and IIb (*p* = 0.0001) (Figure 3f). Although *Myh7* expression was upregulated in Unc5a siRNA‐treated myoblasts on day 1 (*p* = 0.040 [Unc5a si vs. Unc5b si], *p* = 0.045 [Unc5a si vs. Unc5c si], *p* = 0.019 [Unc5a si vs. Neo1 si]) and day 3 (*p* = 0.00038 [Unc5a si vs. Control si], *p* = 0.00011 [Unc5a si vs. Unc5b si], *p* = 0.00060 [Unc5a si vs. Unc5c si], *p* = 0.00005 [Unc5a si vs. Neo1 si]) (Figure 3b), the slow‐type MyHC protein level has not changed (Figure 3f). The total MyHC level has been downregulated by UNC5A knockdown (*p* = 0.0008) (Figure 3f). UNC5C knockdown did not affect the protein levels of any MyHC isoform, except for a significant reduction in *Myh4* expression on day 1, compared with the control siRNA, Unc5A siRNA, and Unc5b siRNA groups (*p* = 0.00033 [Unc5c si vs. Control si], *p* = 0.00390 [Unc5c si vs. Unc5a si], *p* = 0.01721 [Unc5c si vs. Unc5b si]) (Figure 3e,g). Neogenin knockdown significantly reduced the levels of *Myh4* during the middle stage of myogenic differentiation (Day 1: *p* = 0.0117 [Neo1 si vs. Control si]; Day 3: *p* = 0.0406 [Neo1 si vs. Unc5a si], *p* = 0.0475 [Neo1 si vs. Unc5b si]), and also decreased the levels of *Myh7* (*p* = 0.0013 [Neo1 si vs. Control si]) and *Myh2* (*p* = 0.0102 [Neo1 si vs. Control si]) on day 5 (Figure 3c–e). The protein levels of all MyHC isoforms, including slow‐type MyHC, may have decreased compared with the control siRNA group (Slow MyHC: *p* = 0.050; Fast MyHC: *p* = 0.054; MyHC IIa: *p* = 0.083; MyHC IIx: *p* = 0.026) (Figure 3g). UNC5B knockdown resulted in significantly higher levels of *Myh2*, *Myh1*, and *Myh4* on day 5 (*Myh2*: *p* = 0.0002 [Unc5b si vs. Unc5a si], *p* = 0.0008 [Unc5b si vs. Unc5c si], *p* = 0.0002 [Unc5b si vs. Neo1 si]; *Myh1*: *p* = 0.0011 [Unc5b si vs. Unc5a si], *p* = 0.0043 [Unc5b si vs. Unc5c si], *p* = 0.0011 [Unc5b si vs. Neo1 si]; *Myh4*: *p* = 0.0290 [Unc5b si vs. Unc5a si]) (Figure 3c–e), whereas *Myh7* showed a significant reduction (*p* = 0.0349 [Unc5b si vs. Control si]). Unlike other netrin receptors, both fast MyHC and slow MyHC protein levels were elevated along with total MyHC (Fast MyHC: *p* = 0.0372; Slow MyHC: *p* = 0.0125; Total MyHC: *p* = 0.0423) (Figure 3f). Upon these results, we predicted that UNC5A knockdown particularly affects fast‐type MyHC synthesis.

We also performed the knockdown experiments of netrin receptors in EDL and soleus myoblasts during myogenic differentiation (Figure 4a). The knockdown of each receptor markedly decreased the respective receptor levels in both EDL (*Unc5a*: *p* = 0.0231; *Unc5b*: *p* = 0.0002; *Unc5c*: *p* = 0.0805; *Neo1*: *p* = 0.0043) and soleus myoblasts (*Unc5b*: *p* = 0.0035; *Unc5c*: *p* = 0.0003; *Neo1*: *p* = 0.0024), similar to those observed in satellite cell‐derived myoblasts (Figure 3b–e). In these experiments, we confirmed that the expression levels of *Myh2* and *Myh4* in control siRNA‐transfected EDL myoblasts were significantly higher than those in soleus myoblasts (*Myh2*: *p* = 0.0482409; *Myh4*: *p* = 0.0000084) (Figure 4g,i). Consistent with the results in Figure 3b, UNC5A knockdown in EDL myoblasts increased *Myh7* expression (*p* = 0.0568 [Unc5a si vs. Control si]), and decreased *Myh2* (*p* = 0.0014 [Unc5a si vs. Control si], *p* = 0.0038 [Unc5a si vs. Unc5b si]), *Myh1* (*p* = 0.0074 [Unc5a si vs. Control si], *p* = 0.0235 [Unc5a si vs. Unc5b si]), and *Myh4* (*p* = 0.0005 [Unc5a si vs. Control si]) expression on the post‐differentiation 5 d (Figure 4f–i). UNC5A knockdown in soleus myoblasts also significantly reduced *Myh2* (*p* = 0.0014 [Unc5a si vs. Control si], *p* = 0.0023 [Unc5a si vs. Unc5b si]), *Myh1* (*p* = 0.0334 [Unc5a si vs. Unc5b si]), *and Myh4* expression (*p* = 0.0041 [Unc5a si vs. Control si]) (Figure 4g–i). In contrast to the results shown on Day 5 post‐differentiation in Figure 3e, the knockdown of UNC5B or UNC5C significantly inhibited *Myh4* expression in both EDL and soleus myoblasts (EDL: *p* = 0.0027 [Unc5b si vs. Control si], *p* = 0.0004 [Unc5c si vs. Control si]; soleus: *p* = 0.0297 [Unc5b si vs. Control si], *p* = 0.0055 [Unc5c si vs. Control si]) (Figure 4i). UNC5B knockdown also induced *Myh7* upregulation (EDL: *p* = 0.0023 [Unc5b si vs. Control si], *p* = 0.0026 [Unc5b si vs. Unc5c si], *p* = 0.0186 [Unc5b si vs. Neo1 si]; soleus: *p* = 0.0000020 [Unc5b si vs. Control si], *p* = 0.0000006 [Unc5b si vs. Unc5a si], *p* = 0.0000008 [Unc5b si vs. Unc5c si], *p* = 0.0000007 [Unc5b si vs. Neo1 si]) (Figure 4f). Compared with control siRNA‐treated myoblasts, Unc5C siRNA‐transfected myoblasts exhibited a reduction in *Myh2* expression levels in both EDL and soleus myoblasts (EDL: *p* = 0.0204; soleus: *p* = 0.0420) (Figure 4g). The expression of *Myh2* and *Myh4* was decreased by knockdown of neogenin in both EDL myoblasts (*Myh2*: *p* = 0.0214 [Neo1 si vs. Control si]; *Myh4*: *p* = 0.0047 [Neo1 si vs. Control si]) and soleus myoblasts (*Myh2*: *p* = 0.0199 [Neo1 si vs. Control si], *p* = 0.0348 [Neo1 si vs. Unc5b si]; *Myh4*: *p* = 0.0439 [Neo1 si vs. Control si]) (Figure 4g,i), whereas *Myh1* expression was unaffected (Figure 4h). Taken together, these data also suggest that UNC5A has the potency to regulate fast‐twitch myotube formation.

### 3.3 UNC5A knockdown in EDL‐myoblasts exhibited low levels of Sox6, a transcription factor involved in fast‐twitch myofiber formation

Various transcription factors are involved in the formation of fast‐twitch myofibers (Schiaffino et al., 2025; An et al., 2011; Hagiwara et al., 2007; Lee et al., 2015; Meng et al., 2013; Niro et al., 2010; Sadaki et al., 2023; Sakakibara et al., 2014, 2016). In this study, we investigated the involvement of these transcription factors through the knockdown treatment of each netrin receptor in EDL and soleus myoblasts at the onset of myogenic differentiation (Figure 5a). In the control siRNA‐treated group, *Mafa* and *Sox6* were expressed at higher levels in EDL than in soleus myoblasts (*Mafa*: *p* = 0.0433; *Sox6*: *p* = 0.0001) (Figure 5b,g). In EDL myoblasts, *Mafa* expression specifically decreased with UNC5A knockdown (*p* = 0.0555) (Figure 5b), and *Sox6* expression was inhibited with UNC5A or UNC5C knockdown (*p* = 0.0066 [Unc5a si vs. Control si], *p* = 0.0444 [Unc5c si vs. Control si]) (Figure 5g). *Smarcd3* expression decreased with UNC5C knockdown (*p* = 0.0173) (Figure 5h). *Mafb*, *Maf*, and *Six4* expression showed no significant differences, regardless of myoblast origin or netrin receptor knockdown (Figure 5c,d,f). Unexpectedly, *Six1* expression in soleus myoblasts was upregulated with neogenin knockdown (*p* = 0.0119 [Neo1 si vs. Control si], *p* = 0.0012 [Neo1 si vs. Unc5a si], *p* = 0.0070 [Neo1 si vs. Unc5b si], *p* = 0.0212 [Neo1 si vs. Unc5c si]) (Figure 5e), and *Tbx15* expression also increased in EDL myoblasts with the knockdown of UNC5A, UNC5C, or neogenin (*p* = 0.0317 [Unc5a si vs. Control si], *p* = 0.0085 [Unc5c si vs. Control si], *p* = 0.0204 [Unc5c si vs. Unc5b si], *p* = 0.0322 [Neo1 si vs. Control si]) (Figure 5i). Based on these data, we hypothesized that multiple mechanisms may contribute to fast‐type MyHC synthesis via netrin receptors, with UNC5A acting as a consistent regulator of *Sox6* expression.

### 3.4 Elevated fast‐type MyHC level induced by netrin‐1 or netrin‐4 was suppressed under UNC5A knockdown

Given the downregulation of fast‐type MyHC isoforms in UNC5A knockdown‐treated cultures, we investigated whether UNC5A contributes to fast‐twitch myotube formation through the activities of its ligands, netrin‐1 and netrin‐4. Satellite cell‐derived myoblasts were transfected with UNC5A siRNA at the onset of myogenic differentiation, and recombinant netrin‐1 or netrin‐4 protein was added 24 h later to examine the changes in each MyHC isoform levels (Figure 6a). Compared to the control siRNA‐treated group without ligand addition, UNC5A expression was significantly decreased by UNC5A knockdown, regardless of adding netrin‐1 or netrin‐4 (*p* = 0.0295 [Unc5a si vs. Control si], *p* = 0.0279 [Unc5a si + netrin‐1 vs. Control si], *p* = 0.0129 [Unc5a si + netrin‐4 vs. Control si]) (Figure 6b). Consistent with the other experiments, UNC5A knockdown treatment induced *Myh7* expression (*p* = 0.0009) (Figure 6c). Slow MyHC protein band patterns were relatively more evident in the UNC5A knockdown groups, even in the presence of netrin‐1 or netrin‐4, than in the control siRNA‐treated group; nevertheless, the differences did not reach statistical significance (Figure 6g). While *Myh1* expression showed no significant differences after adding netrin‐1 or netrin‐4, or knockdown of UNC5A (Figure 6e), the netrin‐1‐ and netrin‐4‐induced increases in *Myh4* expression were canceled by UNC5A knockdown (*p* = 0.003202 [Control si vs. Control si + netrin‐1], *p* = 0.078430 [Control si vs. Control si + netrin‐4], *p* = 0.045658 [Control si vs. Unc5a si], *p* = 0.023866 [Control si vs. Unc5a si + netrin‐1], *p* = 0.019980 [Control si vs. Unc5a si + netrin‐4], *p* = 0.000003 [Control si + netrin‐1 vs. Unc5a si + netrin‐1], *p* = 0.000045 [Control si + netrin‐4 vs. Unc5a si + netrin‐4]) (Figure 6f). *Myh2* expression was suppressed by Unc5A knockdown, particularly in the presence of recombinant netrin‐4 (*p* = 0.0479 [Control si + netrin‐4 vs. Unc5a si + netrin‐4]) (Figure 6d). The detected protein band patterns of type IIx and IIb isoforms appeared to be downregulated in the Unc5a siRNA‐treated group, even in the presence of recombinant netrin‐1 or netrin‐4; however, no statistically significant differences were observed (Figure 6g). On the contrary, UNC5A knockdown suppressed fast MyHC synthesis, despite supplementation with recombinant netrin‐4 (*p* = 0.0158 [Control si vs. Unc5a si + netrin‐4], *p* = 0.1012 [Control si vs. Unc5a si], *p* = 0.0550 [Control si + netrin‐4 vs. Unc5a si + netrin‐4]). These data suggest that UNC5A acts as a key receptor mediating netrin‐ligand‐induced fast‐twitch myotube formation.

### 3.5 Knockdown of each netrin receptor inhibits Myf6 expression

To further investigate the role of netrin receptors in myogenesis, we performed the knockdown of each netrin receptor at the onset of differentiation in satellite cell‐derived myoblast cultures and examined the expression of mRNAs associated with myogenic transcription factors (*Myod1*, *Myog*, and *Myf6*) and myoblast fusion (*Mymk* and *Mymx*) (Figure 7a). UNC5A siRNA‐treated myoblasts showed significantly higher expression of *Myod1* during the middle stage of myogenic differentiation, compared with other netrin receptor siRNA‐treated cells (Day 1: *p* = 0.0349 [Unc5a si vs. Unc5b si], *p* = 0.0209 [Unc5a si vs. Unc5c si], *p* = 0.0067 [Unc5a si vs. Neo1 si]; Day 3: *p* = 0.0185 [Unc5a si vs. Unc5b si], *p* = 0.0054 [Unc5a si vs. Unc5c si], *p* = 0.0002 [Unc5a si vs. Neo1 si]) (Figure 7b). Likewise, the expression level of *Myf6* in the UNC5A knockdown cultures was not affected by 3 days post‐differentiation (Day 1: *p* = 0.0025 [Unc5a si vs. Unc5b si], *p* = 0.0103 [Unc5a si vs. Unc5c si], *p* = 0.0170 [Unc5a si vs. Neo1 si], Day 3; *p* = 0.0040 [Unc5a si vs. Unc5b si]) (Figure 7d). In contrast, the knockdown treatment of UNC5A resulted in remarkable downregulation in *Myf6* expression at 5 days post‐differentiation, even though neogenin knockdown treatment also inhibited that expression (*p* = 0.000003 [Unc5a si vs. Control si], *p* = 0.000473 [Unc5a si vs. Neo1 si]) (Figure 7d). Neogenin knockdown significantly reduced the expression of *Myod1* (Day 3: *p* = 0.0030 [Neo1 si vs. Control si]), *Myog* (Day 3: *p* = 0.0048 [Neo1 si vs. Control si], *p* = 0.0071 [Neo1 si vs. Unc5a si]), and *Myf6* (Day 3; *p* = 0.0261 [Neo1 si vs. Control si]; Day 5: *p* = 0.0051 [Neo1 si vs. Control si], *p* = 0.0004 [Neo1 si vs. Unc5a si], *p* = 0.0026 [Neo1 si vs. Unc5b si], *p* = 0.0420 [Neo1 si vs. Unc5b si]) (Figure 7b–d). UNC5B or UNC5C knockdown consistently decreased the expression of *Myf6* during the myogenic differentiation phase (Day 1: *p* = 0.009103 [Unc5b si vs. Control si], *p* = 0.040491 [Unc5c si vs. Control si]; Day 3: *p* = 0.001444 [Unc5b si vs. Control si], *p* = 0.021060 [Unc5c si vs. Control si]; Day 5; *p* = 0.000011 [Unc5b si vs. Control si], *p* = 0.000072 [Unc5c si vs. Control si]) (Figure 7d). Intriguingly, the expression levels of *Mymk* and *Mymx* were not remarkably changed by netrin receptor knockdown treatments, while several significant differences in *Mymx* were detected by 3 days post‐differentiation (Day 1: *p* = 0.0238 [Unc5c si vs. Control si], *p* = 0.0152 [Unc5c si vs. Unc5a si]; Day 3: *p* = 0.0201 [Unc5a si vs. Unc5c si], *p* = 0.0022 [Unc5a si vs. Neo1 si]) (Figure 7e,f). Based on these results, UNC5A, UNC5B, UNC5C, and neogenin may also contribute to promoting myogenic differentiation primarily through the regulation of *Myh6* expression.

## 4 DISCUSSION

We previously confirmed that UNC5B, UNC5C, and neogenin mRNAs were clearly expressed in satellite cell‐derived myoblasts 72 h after differentiation (Suzuki et al., 2021); however, the time‐course‐dependent dynamics of netrin receptor expression in myoblasts isolated from soleus and EDL during myogenic differentiation have been unclear. Therefore, this study firstly demonstrated the expression profiles of UNC5A, UNC5B, UNC5C, UNC5D, DCC, and neogenin in myoblasts during myogenic differentiation. We expected that netrin receptors would exhibit high expression in EDL myoblasts because their ligand, netrin‐1, is highly expressed in EDL myoblasts compared to soleus myoblasts and promotes fast‐twitch myotube formation (Suzuki et al., 2021). Although the mRNA expression and protein synthesis of UNC5A, UNC5B, UNC5C, and neogenin were consistently detected during myogenic differentiation, no significant differences were observed between soleus‐ and EDL‐derived myoblasts. Our previous study, which focused on slow‐twitch myotube formation induced by semaphorin 3A ligand, demonstrated that a component of the semaphorin 3A receptor complex, plexin A2, was highly expressed in soleus‐derived myoblasts compared with EDL myoblasts; however, the potent candidates of semaphorin 3A receptor were neuropilin 2‐plexin A3 (Suzuki et al., 2013; Tatsumi et al., 2017). Therefore, within the autonomous myofiber type specification system by multipotent modulators synthesized in myoblasts, we consider that validation based on myoblast origins in the ligand‐synthesis pattern may be more critical than validation based on the receptor‐expression pattern.

Subsequently, we examined the involvement of netrin receptors in myofiber‐type specification. Intriguingly, UNC5A knockdown in satellite cell‐derived myoblasts markedly reduced the protein synthesis of fast MyHC, which recognized all type II isoforms, at the late stage of differentiation, while leaving the expression of slow MyHC isoforms unaffected. A severe impairment of the fast‐twitch myotube fusion was also observed following UNC5A knockdown, in contrast to the other netrin receptor knockdown treatments. Netrin‐1 and netrin‐4, ligands of UNC5A (Finci et al., 2014; Geisbrecht et al., 2003; Qin et al., 2007), are among a family of netrins that induce fast MyHC expression (Maeno et al., 2023; Suzuki et al., 2021). We directly examined whether UNC5A‐mediated formation of fast‐twitch myotubes is facilitated by netrin‐1 and netrin‐4. In myoblasts transfected with control siRNA, treatment with recombinant netrin‐1 or netrin‐4 enhanced the expression of *Myh4*; however, this effect was abolished by UNC5A knockdown, indicating that the inability of netrin‐1 or netrin‐4 to bind to UNC5A prevented *Myh4* induction. These findings confirm that UNC5A regulates fast MyHC (especially *Myh4*) expression through its interaction with netrin‐1 and netrin‐4, thereby promoting fast‐twitch myotube formation.

UNC5B knockdown increased the protein synthesis of both slow and fast MyHC isoforms. Despite the high expression of mRNAs in soleus myoblasts during myogenic differentiation, UNC5C knockdown did not exert any notable effect on MyHC protein synthesis. These results imply that UNC5B and UNC5C may play roles beyond myofiber‐type specification. Further research should therefore focus on their potential roles in maintaining the self‐renewal and proliferative capacity of satellite cells (myoblasts), given the enhanced self‐renewal and proliferative abilities of slow‐twitch compared to fast‐twitch satellite cells (Lagord et al., 1998; Motohashi et al., 2019). Neogenin knockdown consistently reduced both slow and fast MyHC isoforms during myogenic differentiation; this finding supports previous reports indicating that neogenin is essential for myotube formation itself (Kang et al., 2004). In this study, we performed the single knockdown treatment of each receptor. Therefore, in our further study, we will perform double and triple knockdown treatments of UNC5B, UNC5C, and neogenin, excluding UNC5A, in myoblasts to investigate whether they contribute to the autonomous determination of fast‐ or slow‐twitch myotubes.

We investigated how UNC5A mediates the expression and synthesis of fast‐twitch MyHCs based on the levels of transcription factors involved in fast‐twitch myofiber formation, including MafA (*Mafa*), MafB (*Mafb*), Maf (c‐Maf; *Maf*), Six1 (*Six1*), Six4 (*Six4*), Sox6 (*Sox6*), Baf60c (*Smarcd3*), and Tbx15 (*Tbx15*) (Hagiwara et al., 2007; Lee et al., 2015; Meng et al., 2013; Niro et al., 2010; Sadaki et al., 2023; Sakakibara et al., 2016). Control siRNA‐treated myoblasts highly expressed *Mafa* and *Sox6* in EDL compared to soleus myoblasts. This result suggested that these transcription factors are important determinants of fast‐twitch myotube type even in our experimental model, following the previous reports. Notably, the knockdown of UNC5A in EDL myoblasts reduced the expression of *Mafa* and *Sox6*. Large Mafs (MafA, MafB, and Maf) are crucial for the formation of type IIb myofibers (Sadaki et al., 2023). Sadaki et al. (2023) revealed that single knockouts of MafA, MafB, or Maf did not affect the fiber type profile of adult mouse muscles, whereas triple knockouts resulted in the complete loss of type IIb myofibers. We observed a tendency for only *Mafa* expression to decrease upon UNC5A knockdown, making it unlikely that the decreased MyHC IIb expression was solely due to decreased MafA expression. In contrast, Sox6 suppressed the formation of slow‐twitch myofibers and promoted the formation of fast‐twitch myofibers (An et al., 2011; Hagiwara et al., 2007; Jackson et al., 2015; Zhang et al., 2022). In our study, UNC5A knockdown in satellite cell‐derived myoblasts and EDL myoblasts increased the expression of *Myh7* and significantly decreased the expression of *Myh4* during myogenic differentiation, along with that of *Sox6*. Overexpression of MafA in myotubes does not alter Sox6 expression (Sadaki et al., 2025), and Sox6 expression is increased in Myf6‐knockout satellite cells (Lazure et al., 2020), suggesting that the reduction in Sox6 expression caused by UNC5A knockdown is unlikely to be driven by the decreased expression of MafA or Myf6/MRF4. Therefore, previous observations and the data of this study suggest that UNC5A regulates the formation of fast‐twitch myotubes via Sox6 expression; we propose that the novel signaling pathway comprising netrins → UNC5A → Sox6 ⊣ slow MyHC plays a key regulatory role in fast‐twitch myotube formation.

We also conducted siRNA‐mediated knockdown experiments for each netrin receptor to determine their involvement in the myogenic differentiation of myoblasts. Suppression of UNC5A, UNC5B, UNC5C, or neogenin morphologically inhibited the formation of both fast and slow MyHC‐positive myotubes. These results suggested that netrin receptors may play a role not only in specifying myofiber types but also in the fundamental process of myotube formation. Notably, *Myod1*, *Myog*, and *Myf6* expression was consistently reduced in neogenin‐knockdown myoblasts. Neogenin, a multifunctional receptor for various ligands, including netrin‐1, netrin‐3, BMP2, and RGMa, regulates myogenic differentiation through multiple signaling molecules, such as RhoA, FAK, and ERK, and modulates the expression and transcriptional activity of the myogenic bHLH transcription factors MyoD and Myogenin (Bae et al., 2009; do Carmo Costa et al., 2021; Hagihara et al., 2011; Kang et al., 2004; Suzuki et al., 2021; Wang et al., 2019). Considering that the transcription of Myomaker and Myomixer [membrane proteins essential for myoblast fusion and myotube formation (Bi et al., 2017; Millay et al., 2013, 2014)] is promoted by MyoD and Myogenin (Ganassi et al., 2018; Millay et al., 2014; Takei et al., 2017; Zhang et al., 2020), neogenin seems to promote myotube formation by enhancing the expression and transcriptional activity of MyoD and Myogenin. However, in this study, we did not detect any effects of netrin receptor knockdown treatment, including neogenin, on *Mymk* and *Mymx* expression, which is inconsistent with our previous findings that netrin‐4 knockdown reduced the expression of both *Mymk* and *Mymx*, along with the protein synthesis of MyoD and Myogenin (Maeno et al., 2023). Therefore, we are currently focusing on changes in *Mymk* and *Mymx* expression at later stages, such as myogenic maturation, following netrin receptor knockdown treatment. Furthermore, we are investigating additional potential candidates for novel regulatory factors in myotube fusion and formation stimulated by netrin‐receptor‐related signals, using omics approaches such as RNA sequencing. The knockdown of UNC5A led to a sharp decrease in *Myf6* expression during the late stages of myogenic differentiation. Since Myf6/MRF4 is uniquely expressed in fully differentiated myofibers (Lazure et al., 2020), the suppression of netrin receptor expression likely inhibits the maturation of myotubes. In contrast, Myf6/MRF4 does not contribute to myotube maturation but instead prevents the depletion of satellite cells by inhibiting their differentiation (Lazure et al., 2020; Moretti et al., 2016). Therefore, it is unlikely that the inhibition of myotube formation is directly caused by reduced Myf6/MRF4 expression in UNC5A‐, UNC5B‐, or UNC5C‐knockdown treatments. Further studies are required to determine the molecular mechanisms through which UNC5A, UNC5B, and UNC5C contribute to myotube formation.

## 5 CONCLUSION

In this study, we elucidated the roles of a netrin receptor UNC5A expressed in myoblasts throughout the myogenic differentiation and the myotube formation process. This study demonstrated that UNC5A played a key role in fast‐twitch myotube formation under the regulation of netrin‐1 and netrin‐4 ligands, suggesting that myoblasts possess a novel autonomous system that promotes fast‐twitch myotube formation via autocrine‐paracrine‐dependent mechanisms.

## AUTHOR CONTRIBUTIONS

Takahiro Maeno and Tomoki Ushijima designed the experiments and performed most of the experimental work in this study; Koichi Ojima, Yohei Ogawa, Sayuki Hayashi, Hikaru Imakyure, Rika Osaki, Ryuki Oyama, Aoi Ogawa, Akimasa Takano, Kaoru Mizoguchi, and Takahiro Suzuki assisted Takahiro Maeno and Tomoki Ushijima in each experiment; Takahiro Maeno, Tomoki Ushijima, Koichi Ojima, Issei Yokoyama, Yusuke Komiya, Mako Nakamura, Ryuichi Tatsumi, and Takahiro Suzuki interpreted the experimental data; Koichi Ojima, Issei Yokoyama, Yusuke Komiya, Mako Nakamura, Ryuichi Tatsumi, and Takahiro Suzuki supervised the study; Takahiro Maeno, Ryuichi Tatsumi, and Takahiro Suzuki acquired funding to promote this experimental project; Takahiro Maeno and Takahiro Suzuki wrote the original draft and designed the figures; Koichi Ojima, Mako Nakamura, Ryuichi Tatsumi, and Takahiro Suzuki reviewed the manuscript; Takahiro Suzuki conceived the study, oversaw the research, and edited the manuscript. All authors have read and approved the published version of the manuscript.

## FUNDING INFORMATION

This work was mainly supported by JSPS KAKENHI grants (nos. 19K15959 and 22K05955, and 24K01911 to Takahiro Suzuki and nos. 26660218, 21H02347, and 24K01911 to Ryuichi Tatsumi) and a grant from the Ito Foundation to Takahiro Suzuki. Takahiro Maeno received a JSPS Pre‐Doctoral Research Fellowship for Young Scientists and a Grant‐in‐Aid for JSPS Fellows (no. 22J22522), a scholarship from the ANDO Foundation, a scholarship from the Japan Educational Exchanges and Services, and grant funds for Academic Challenge 2023 from the Robert T. Huang Entrepreneurship Center of Kyushu University and Leave‐a‐Nest Grant.

## CONFLICT OF INTEREST STATEMENT

The authors declare no conflict of interest.

## Supporting information


**Data S1.** Supporting Information.

**Data S2.** Supporting Information.

**Data S3.** Supporting Information.

## Data Availability

The data that support the findings of this study are available from the corresponding author upon reasonable request.
