# Supplementary figures and images for: The uncoordinated‐5 homologue A is a key receptor in netrin‐ligand‐mediated fast‐twitch myotube formation in male mice
Source: Physiol Rep. 2026 Feb 17;14(4):e70788. doi: 10.14814/phy2.70788 (PMC12914085; doi:10.14814/phy2.70788)

Figure S1

Fig. S1A

< UNC5A >

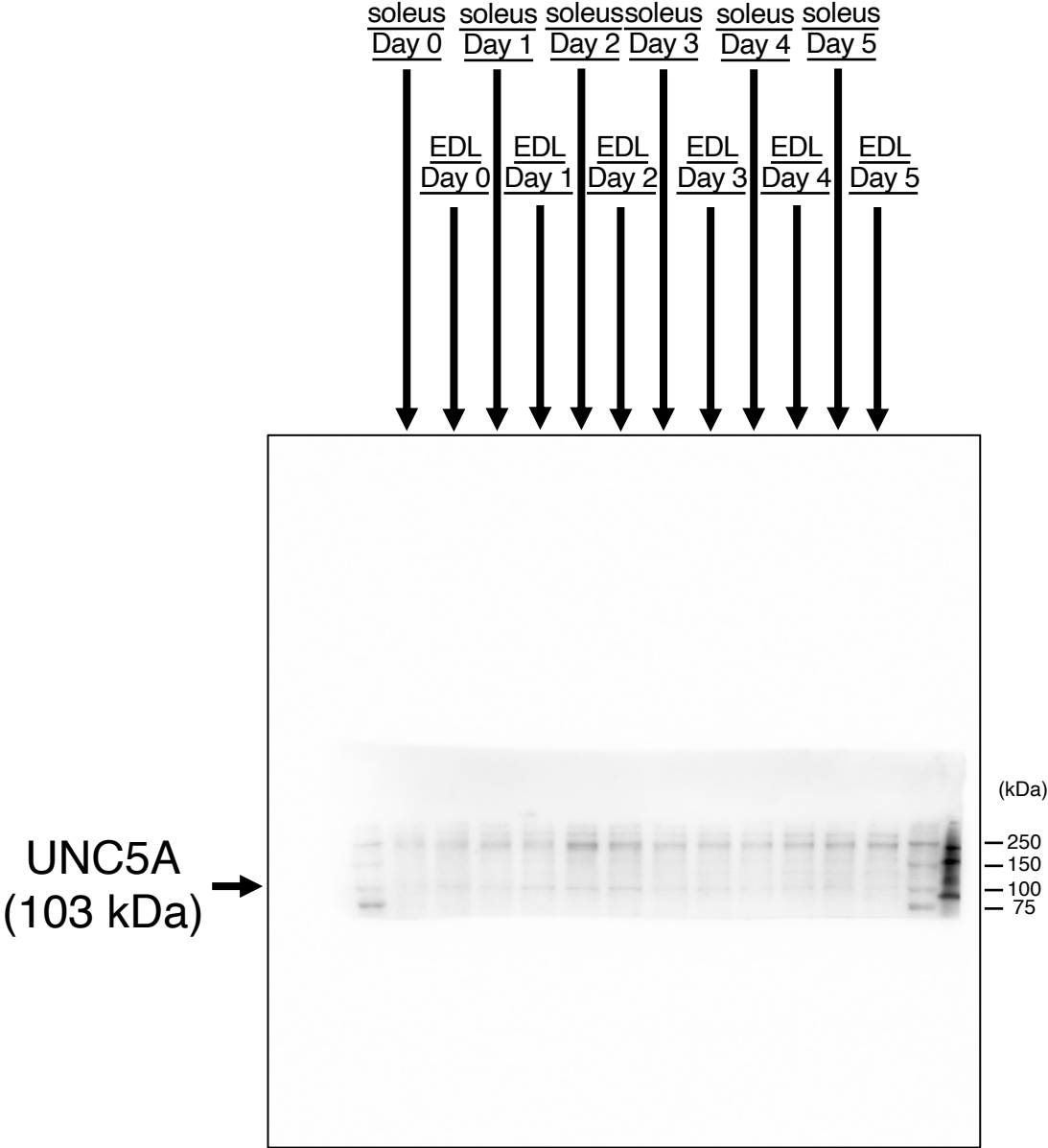

Fig. S1B

< UNC5B >

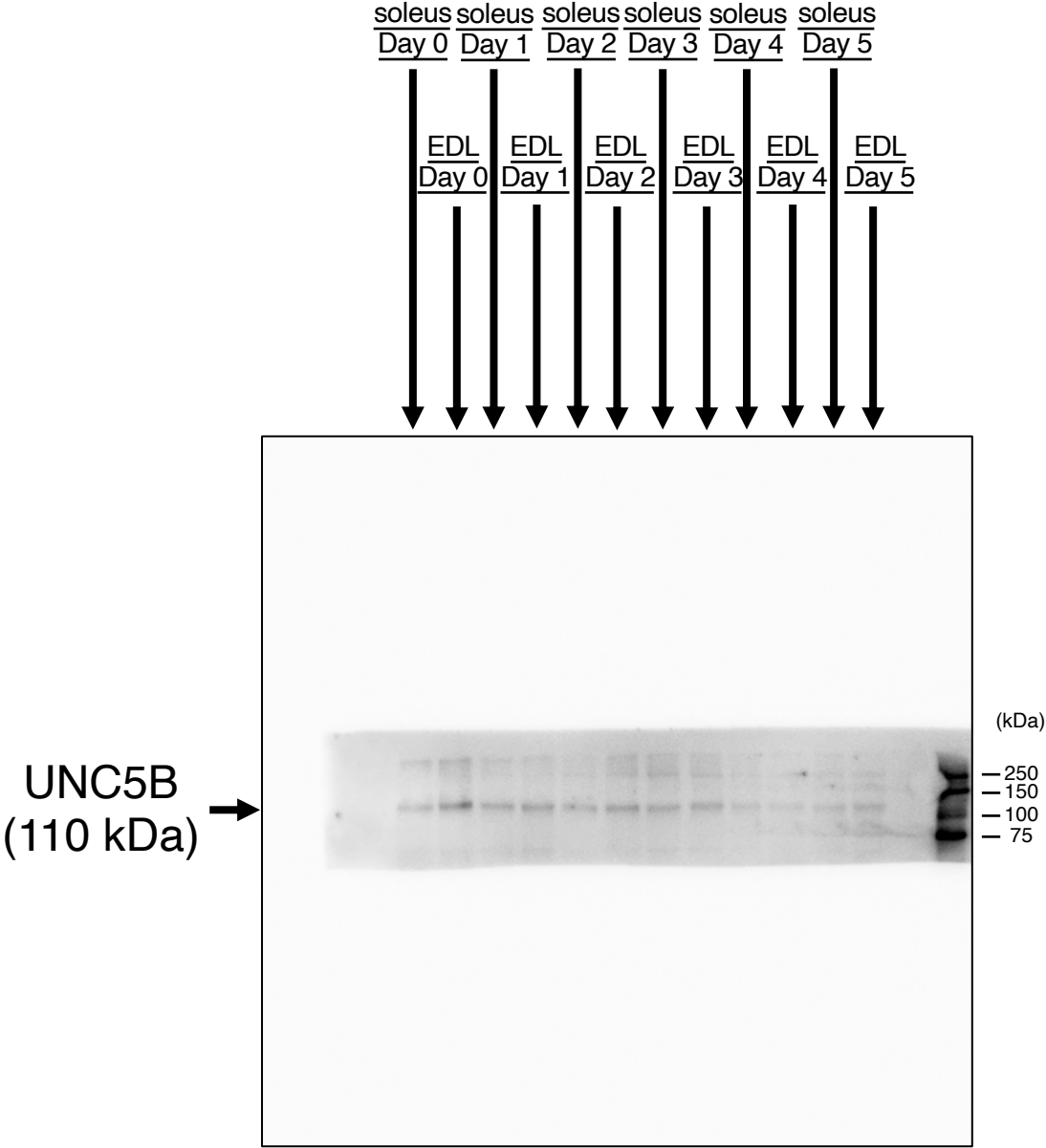

Fig. S1C

< UNC5C >

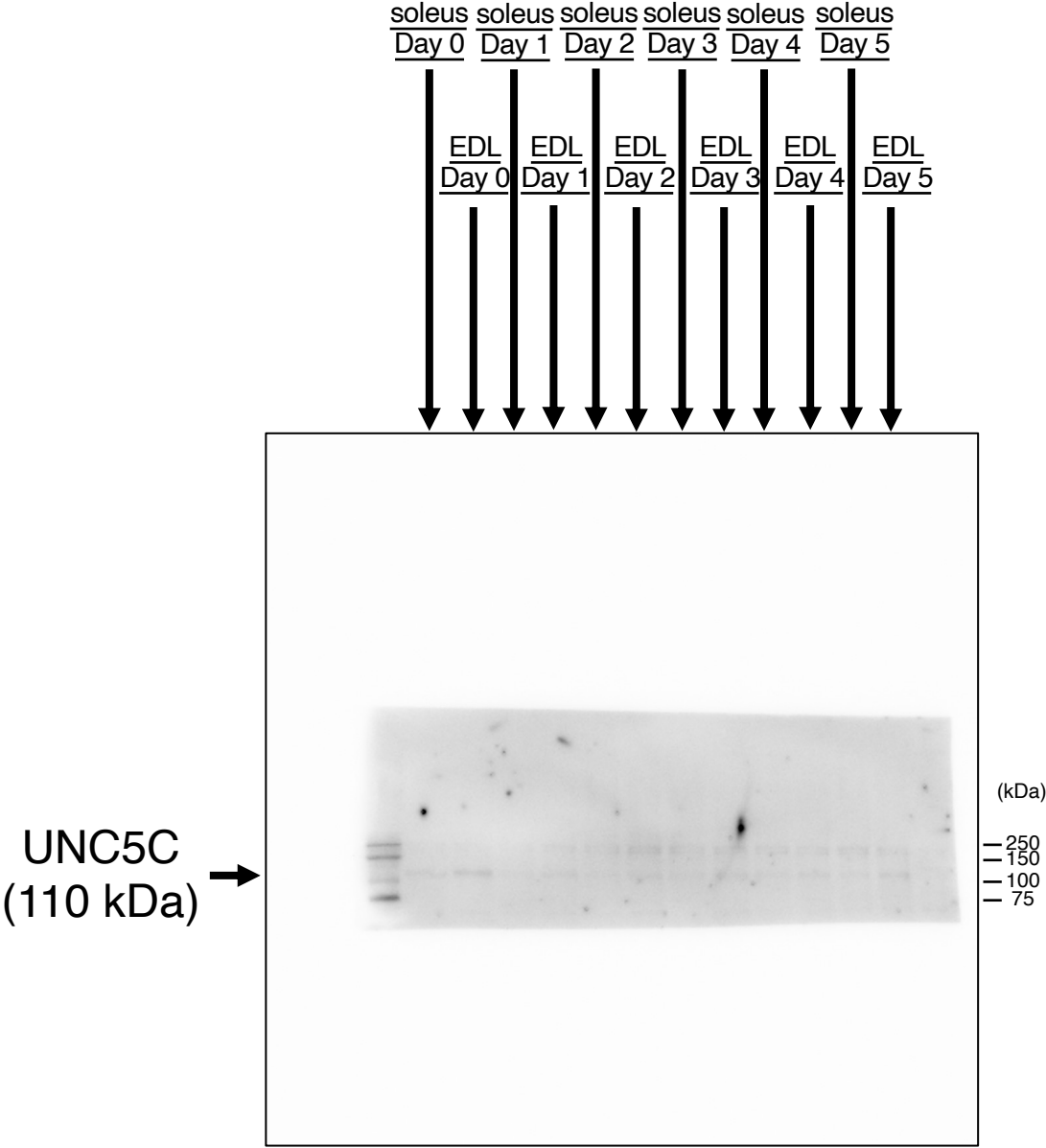

Fig. S1D

< Neogenin >

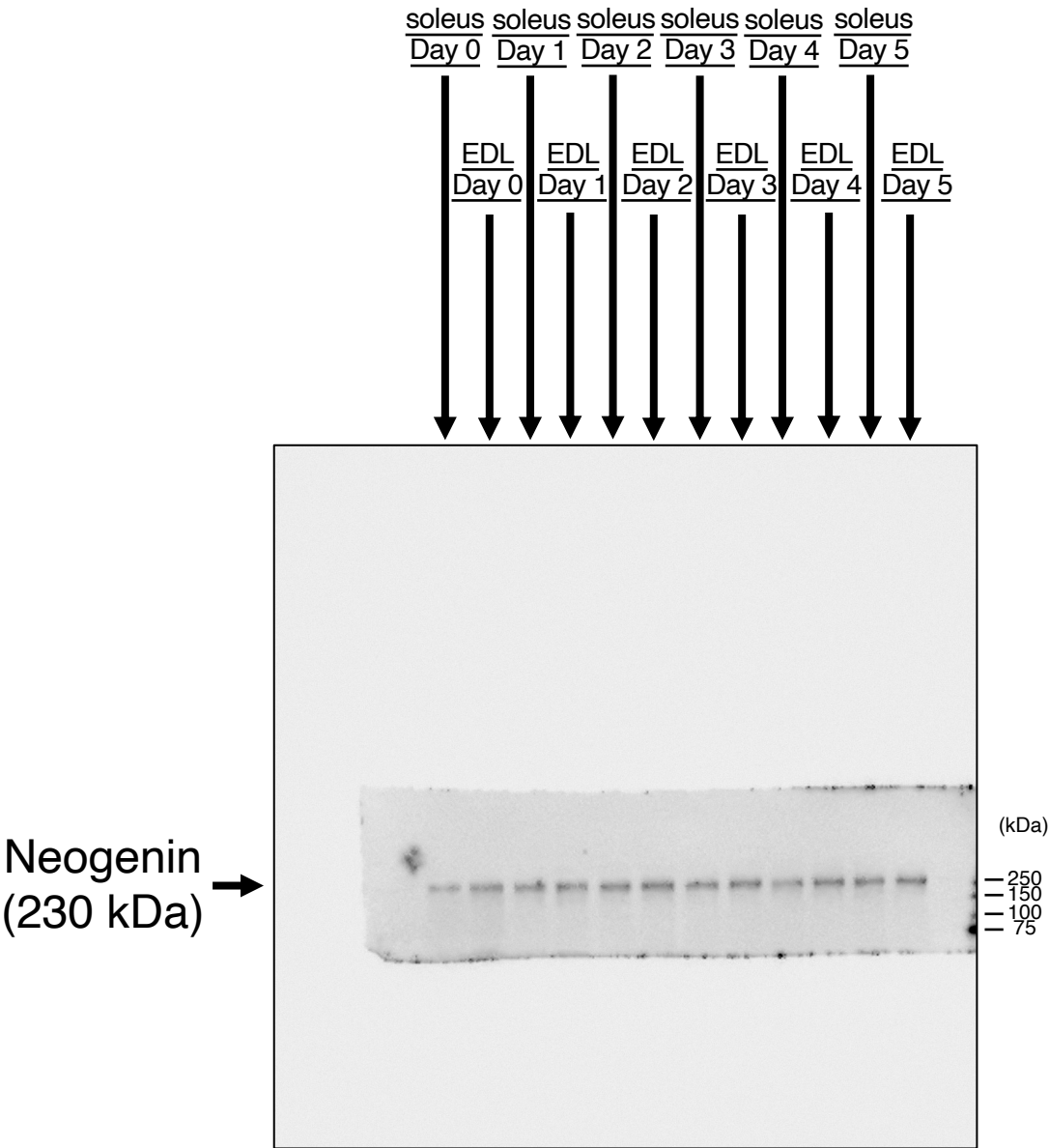

Fig. S1E

<  $\alpha$ -tubulin >

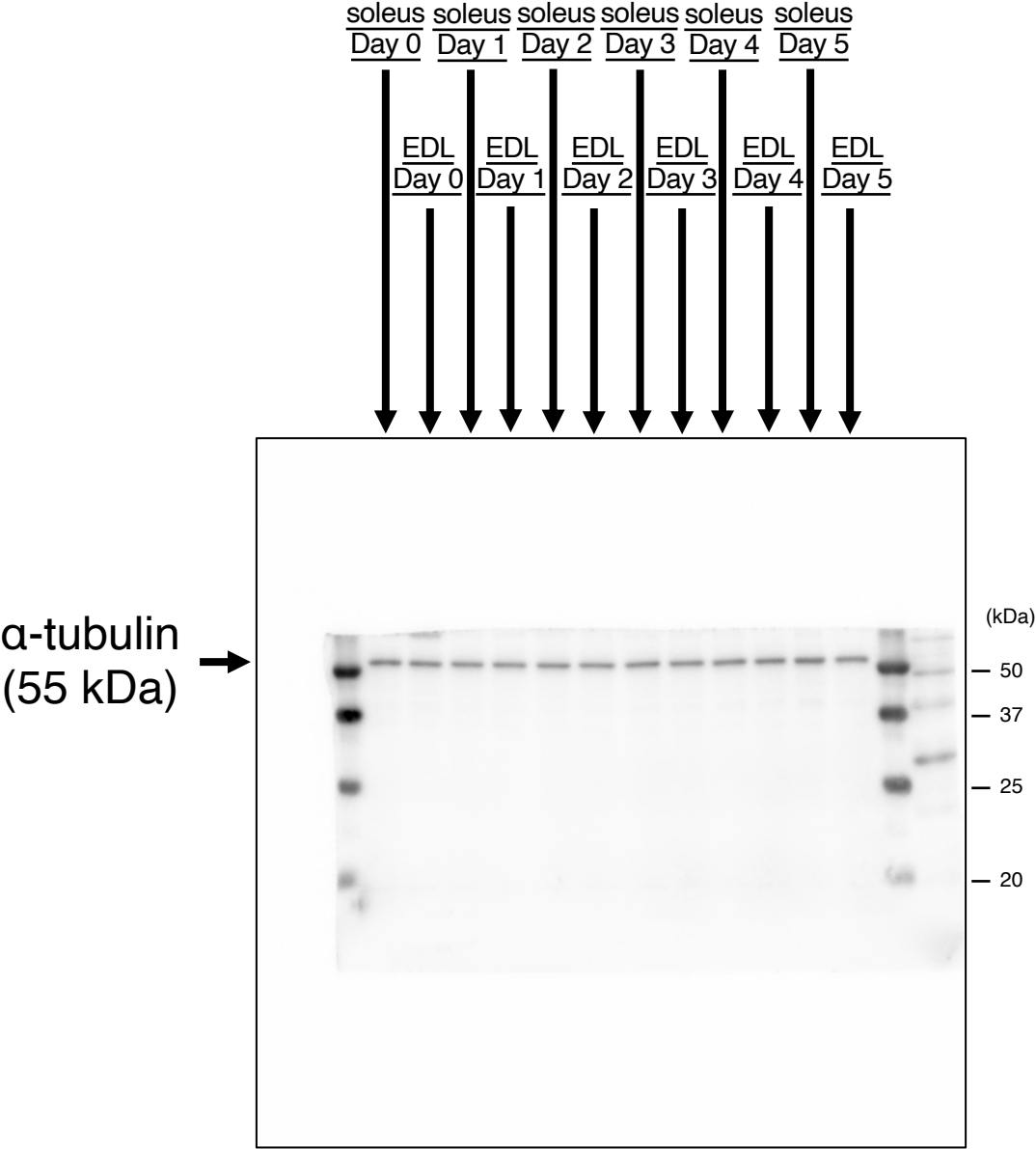

Supplement: Supplementary file 1 — Data S1. Supporting Information. [file PHY2-14-e70788-s002.pdf]

Figure S3

Fig. S3A

< Slow MyHC >

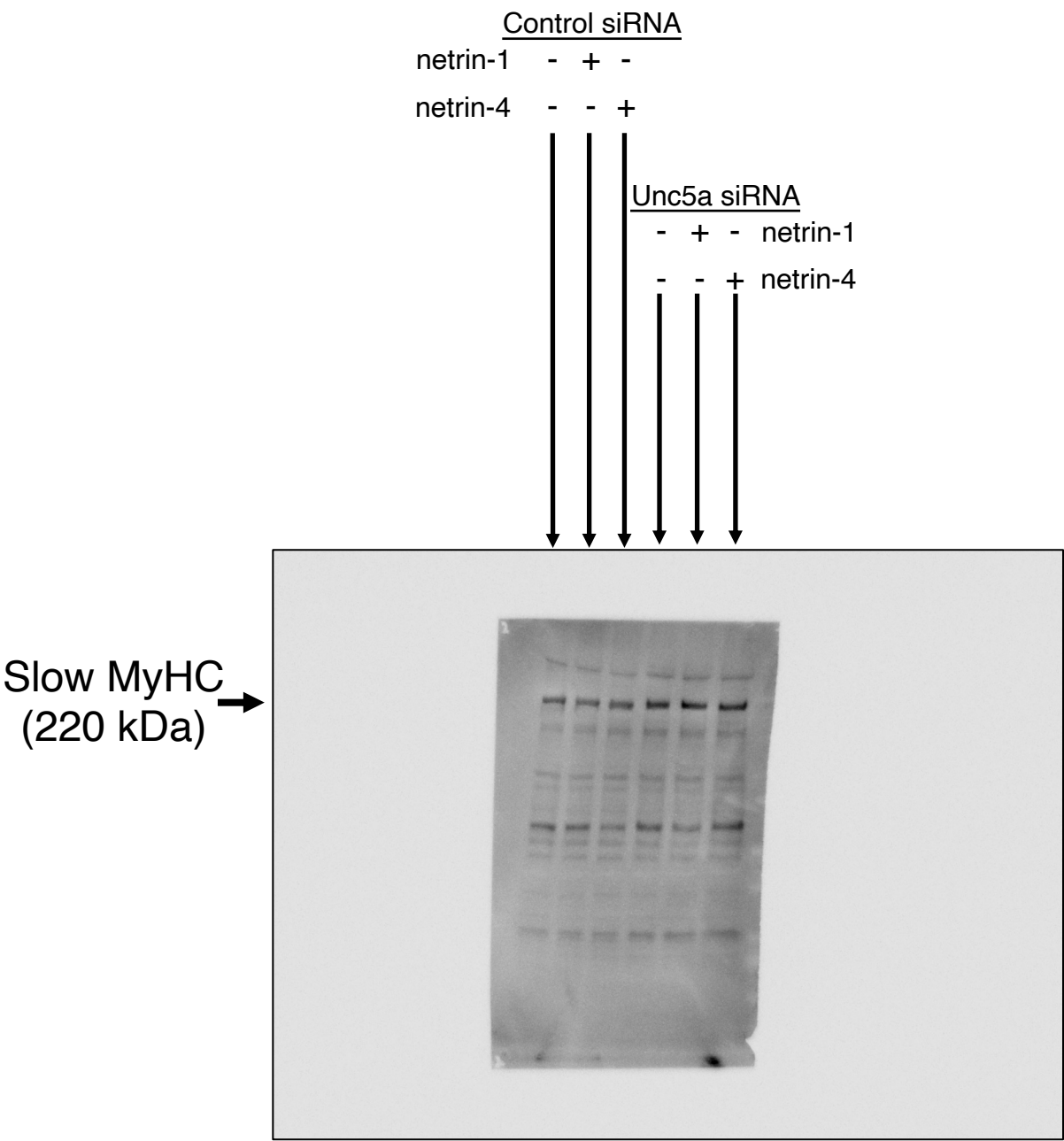

**Fig. S3B**

## < Fast MyHC >

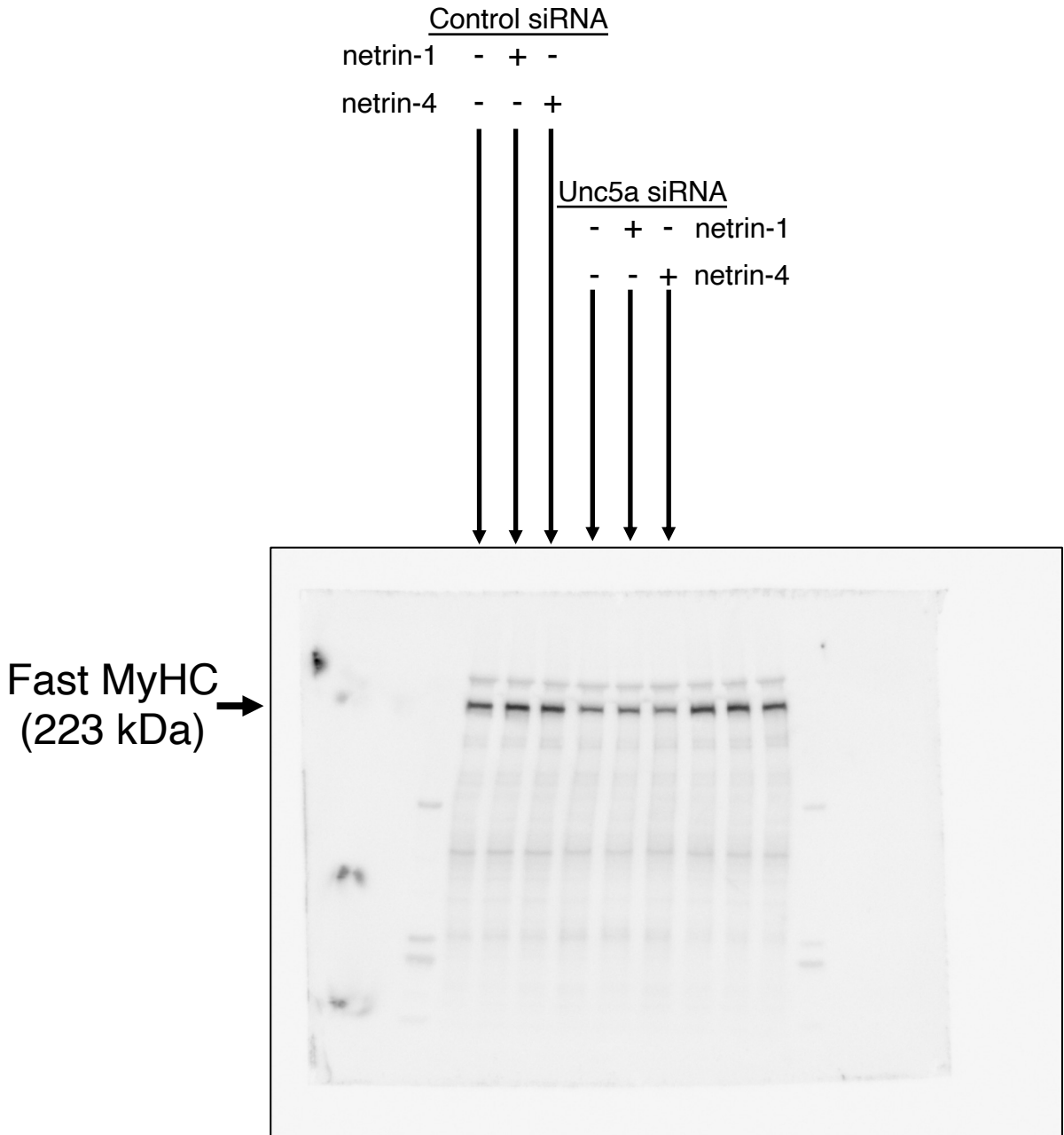

**Fig. S3C**

**< MyHC IIx >**

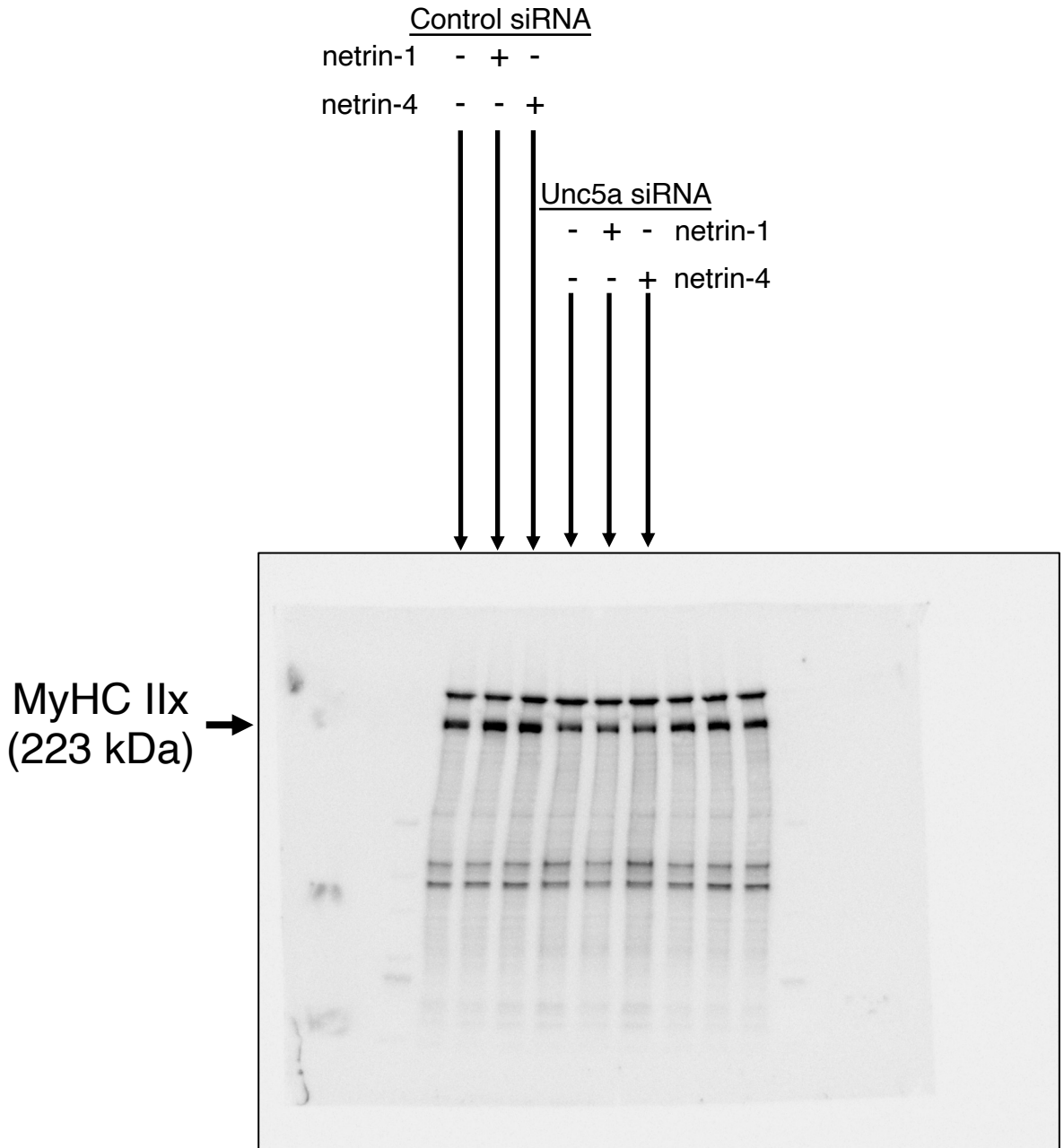

Fig. S3D

< MyHC IIb >

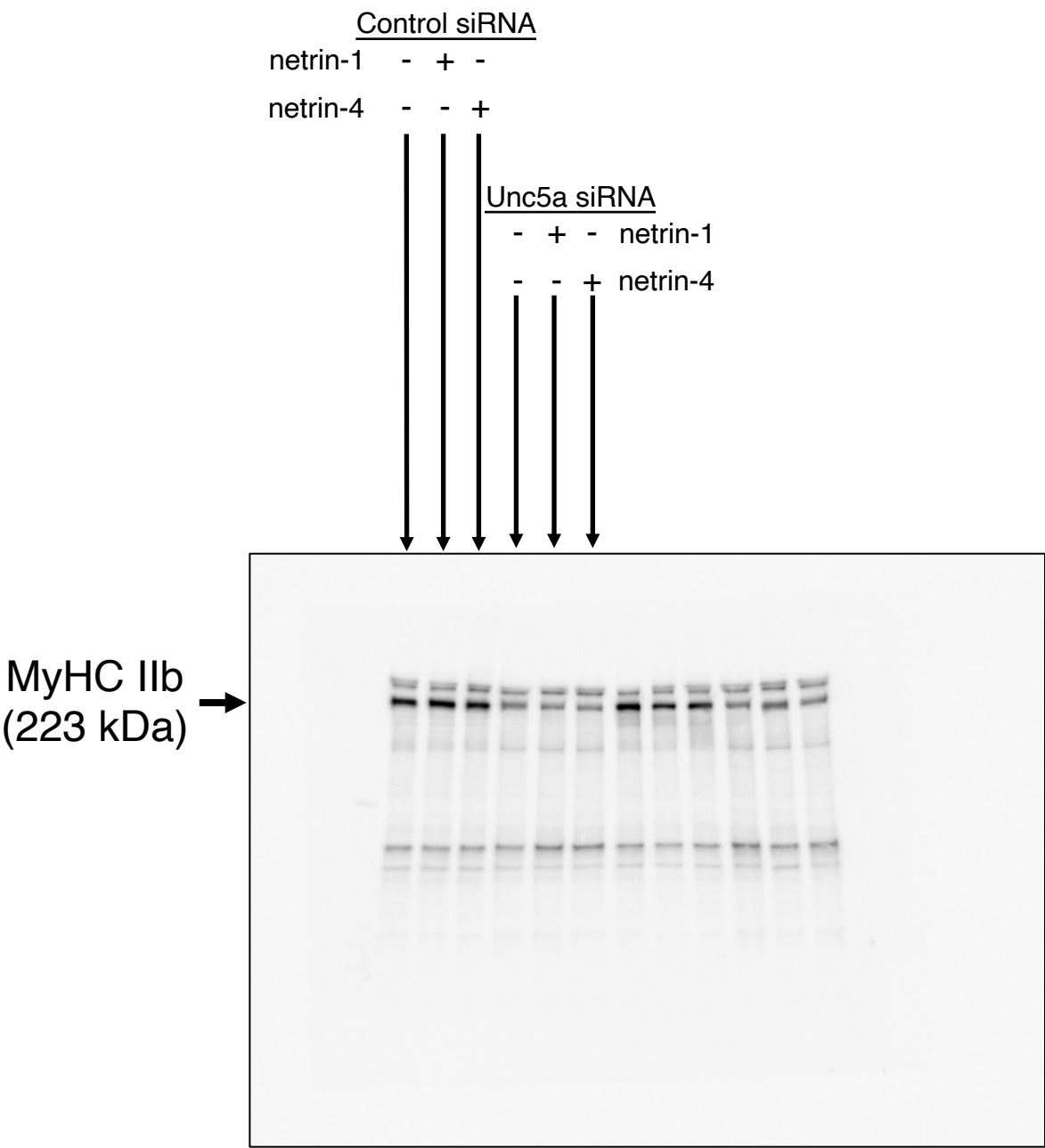

**Fig. S3E**

**< α-tubulin >**

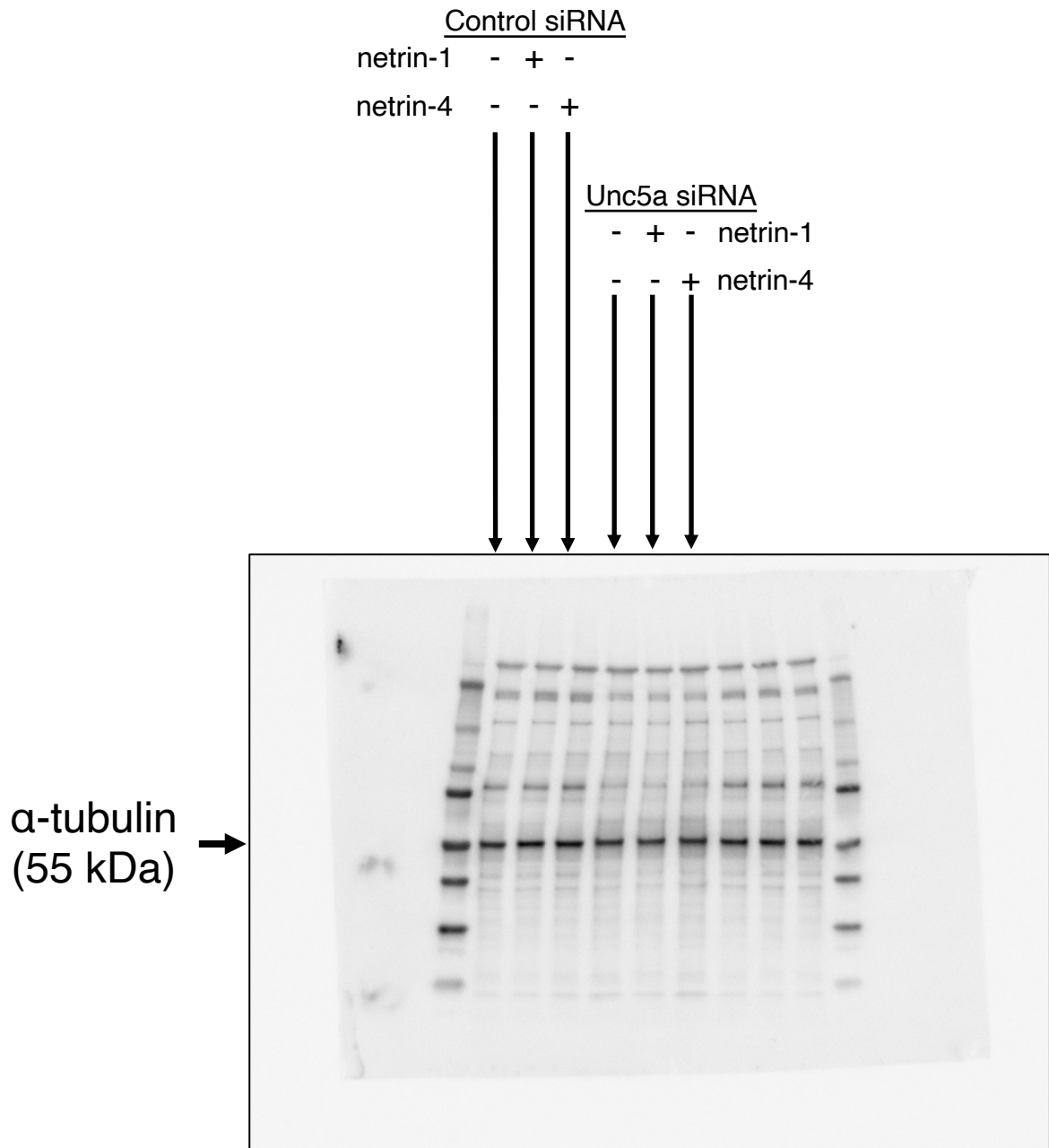

Supplement: Supplementary file 3 — Data S3. Supporting Information. [file PHY2-14-e70788-s003.pdf]
