# Supplementary material for: The uncoordinated‐5 homologue A is a key receptor in netrin‐ligand‐mediated fast‐twitch myotube formation in male mice
Source: Physiol Rep. 2026 Feb 17;14(4):e70788. doi: 10.14814/phy2.70788 (PMC12914085; doi:10.14814/phy2.70788)

Figure S2

Fig. S2A

< Total MyHC >

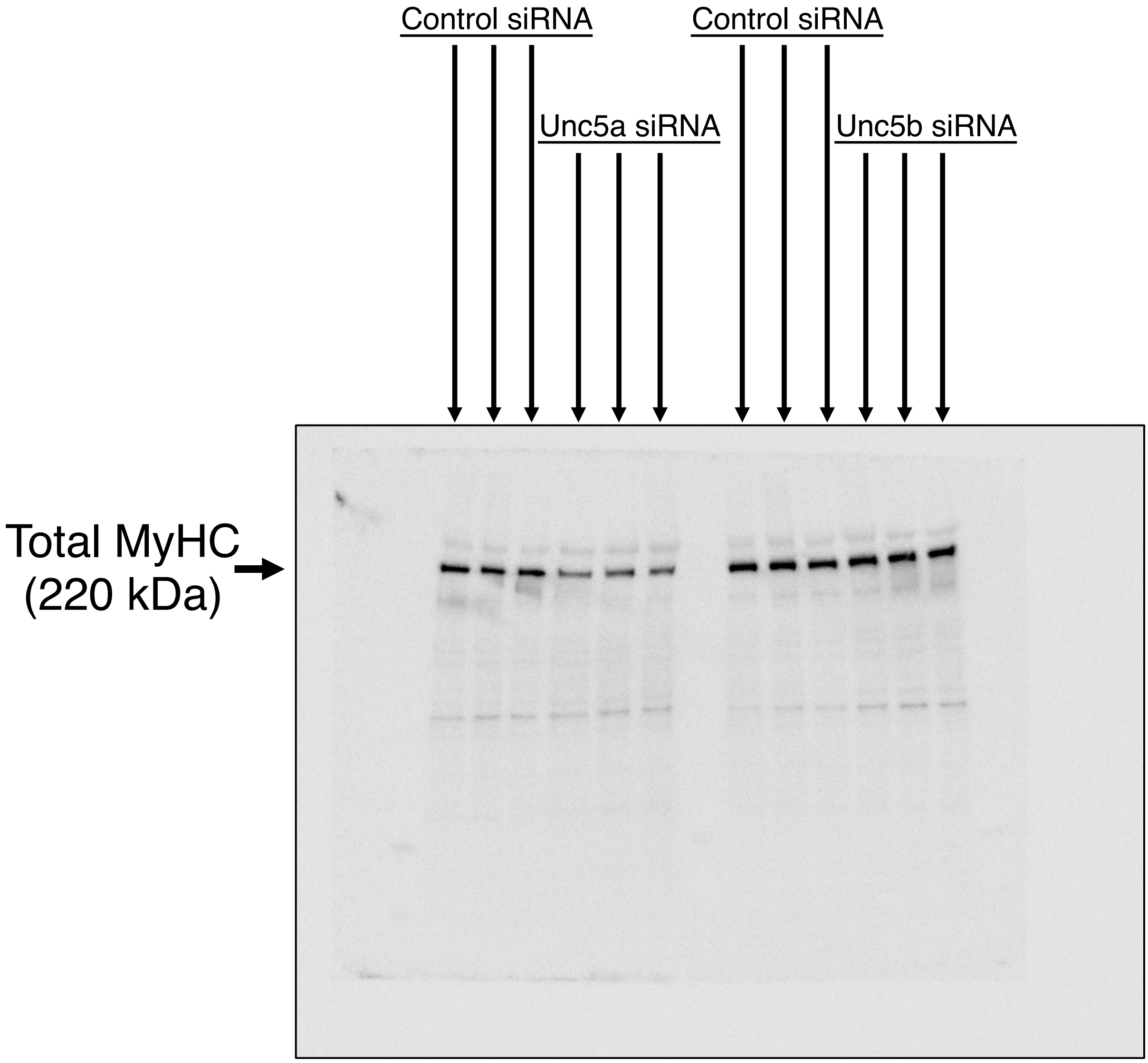

Fig. S2B

< Slow MyHC >

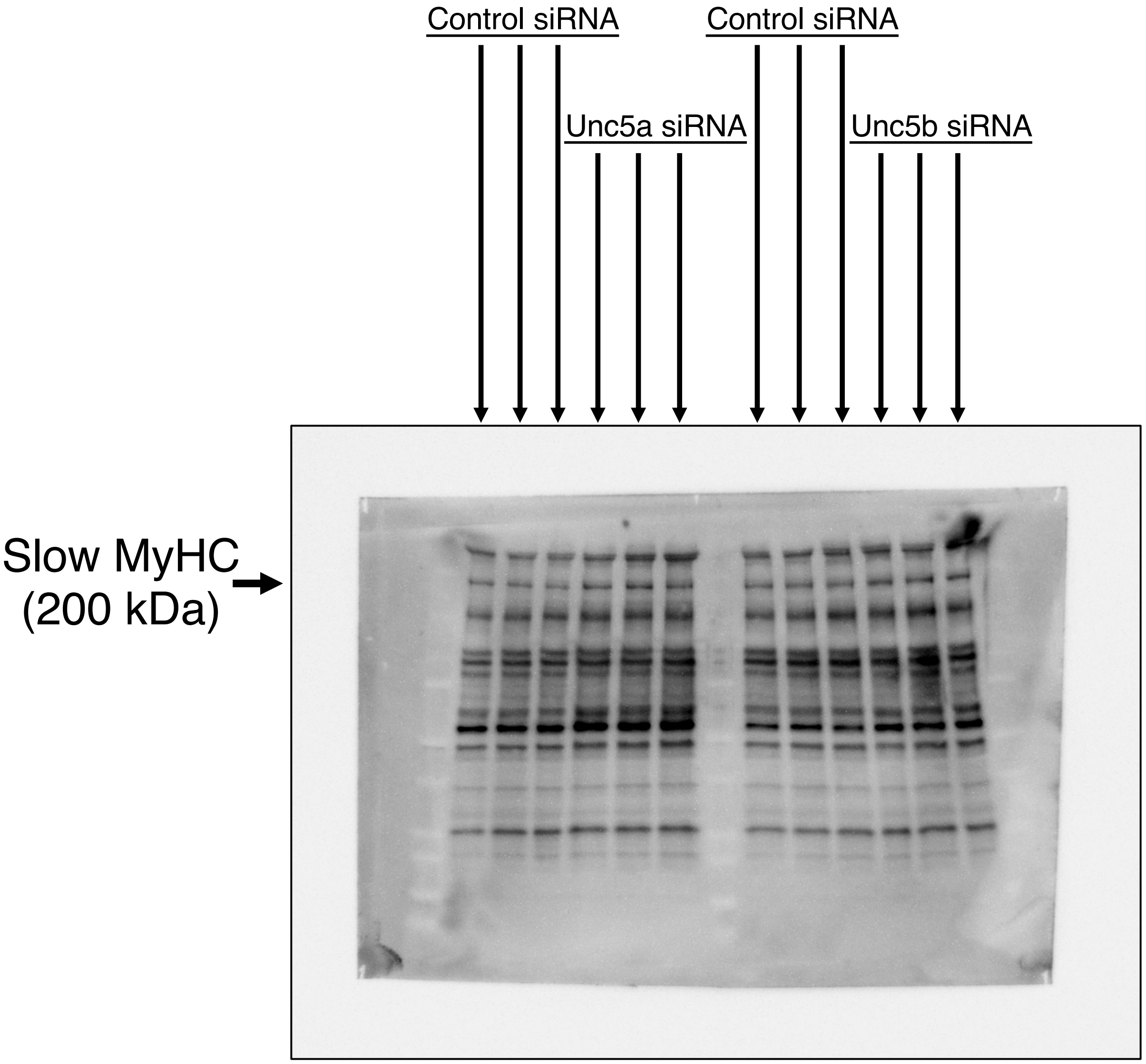

Fig. S2C

< Fast MyHC >

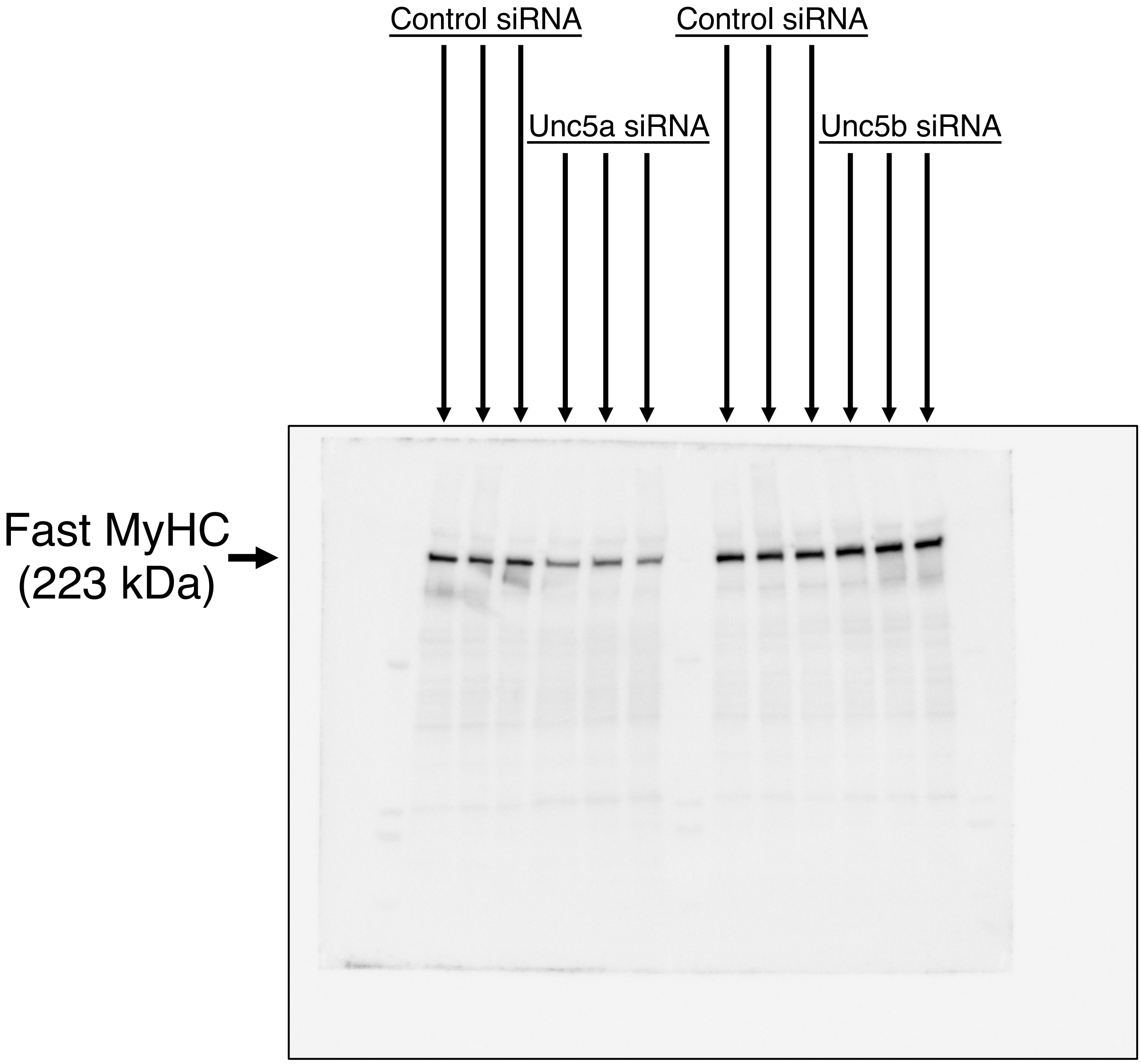

Fig. S2D

< MyHC IIa >

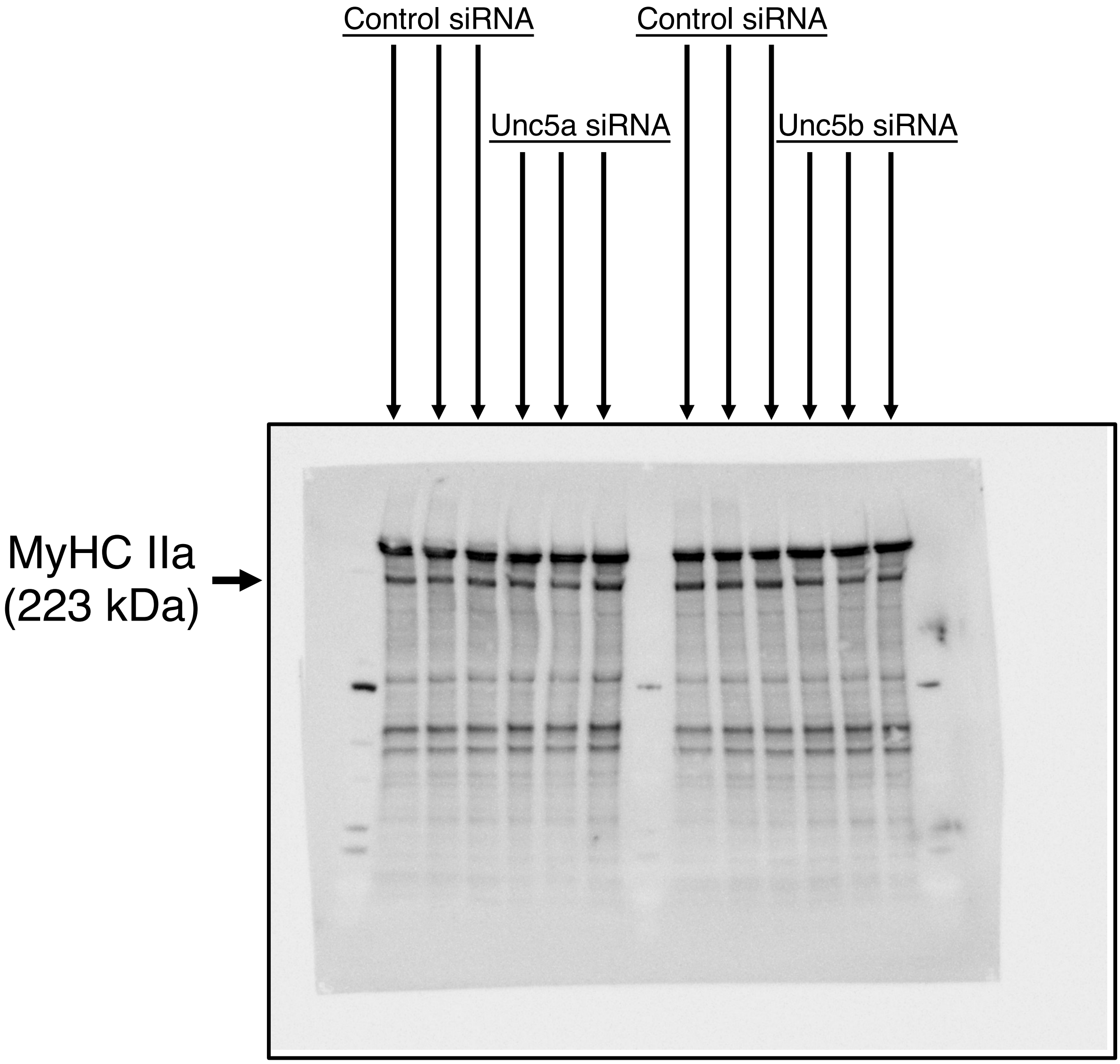

Fig. S2E

< MyHC IIx >

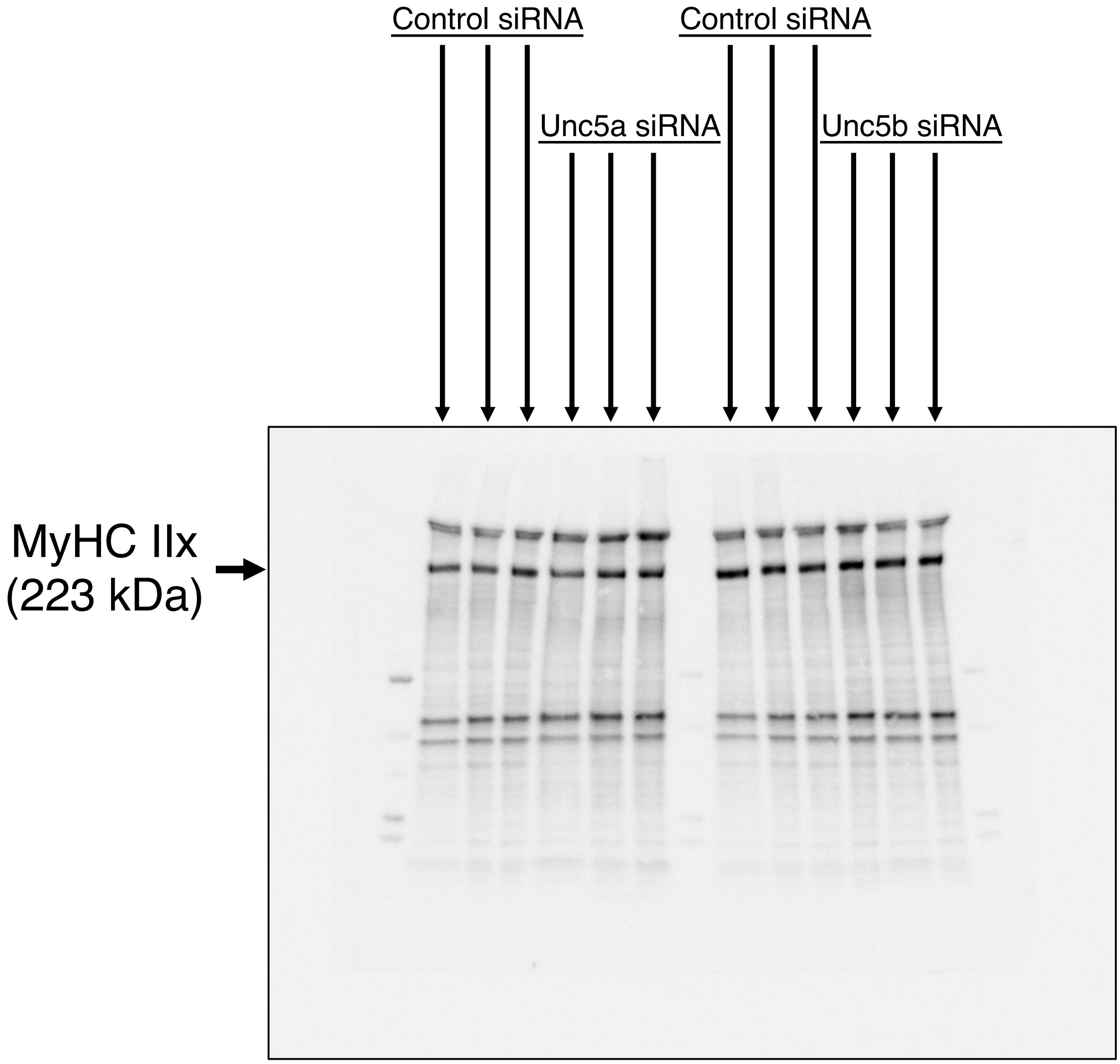

Fig. S2F

< MyHC IIb >

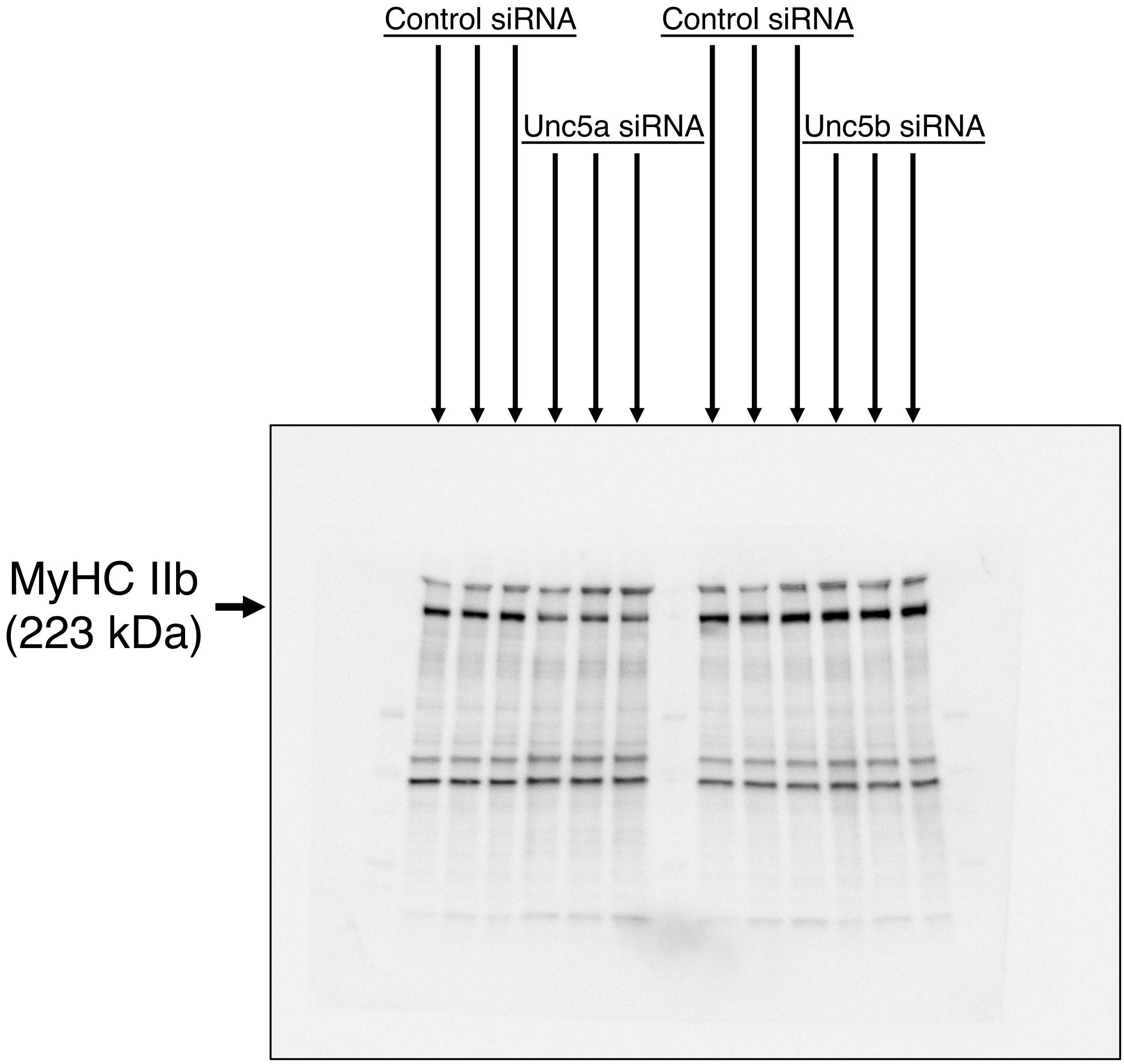

Fig. S2G

< α-tubulin >

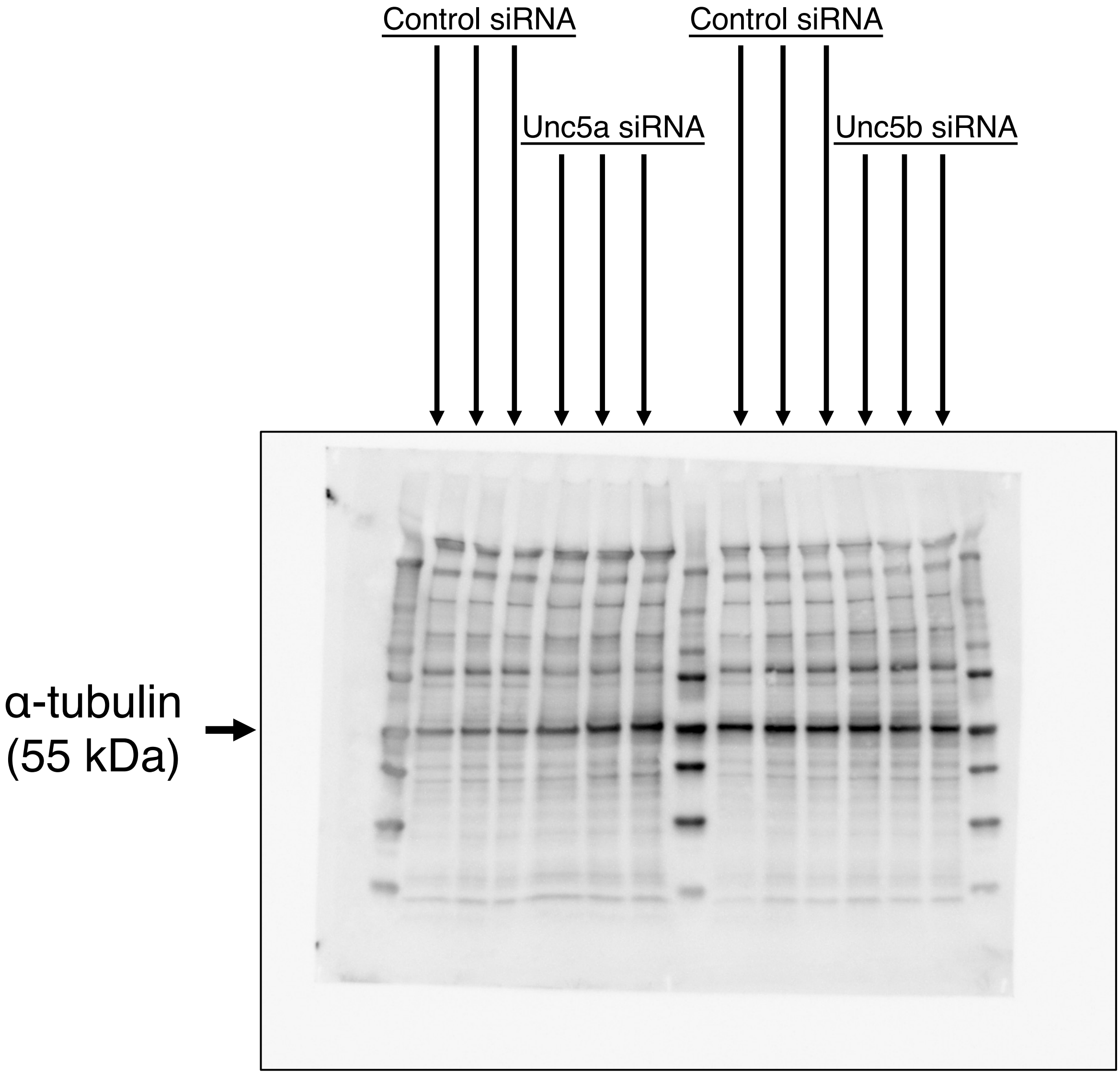

Fig. S2H

< Total MyHC >

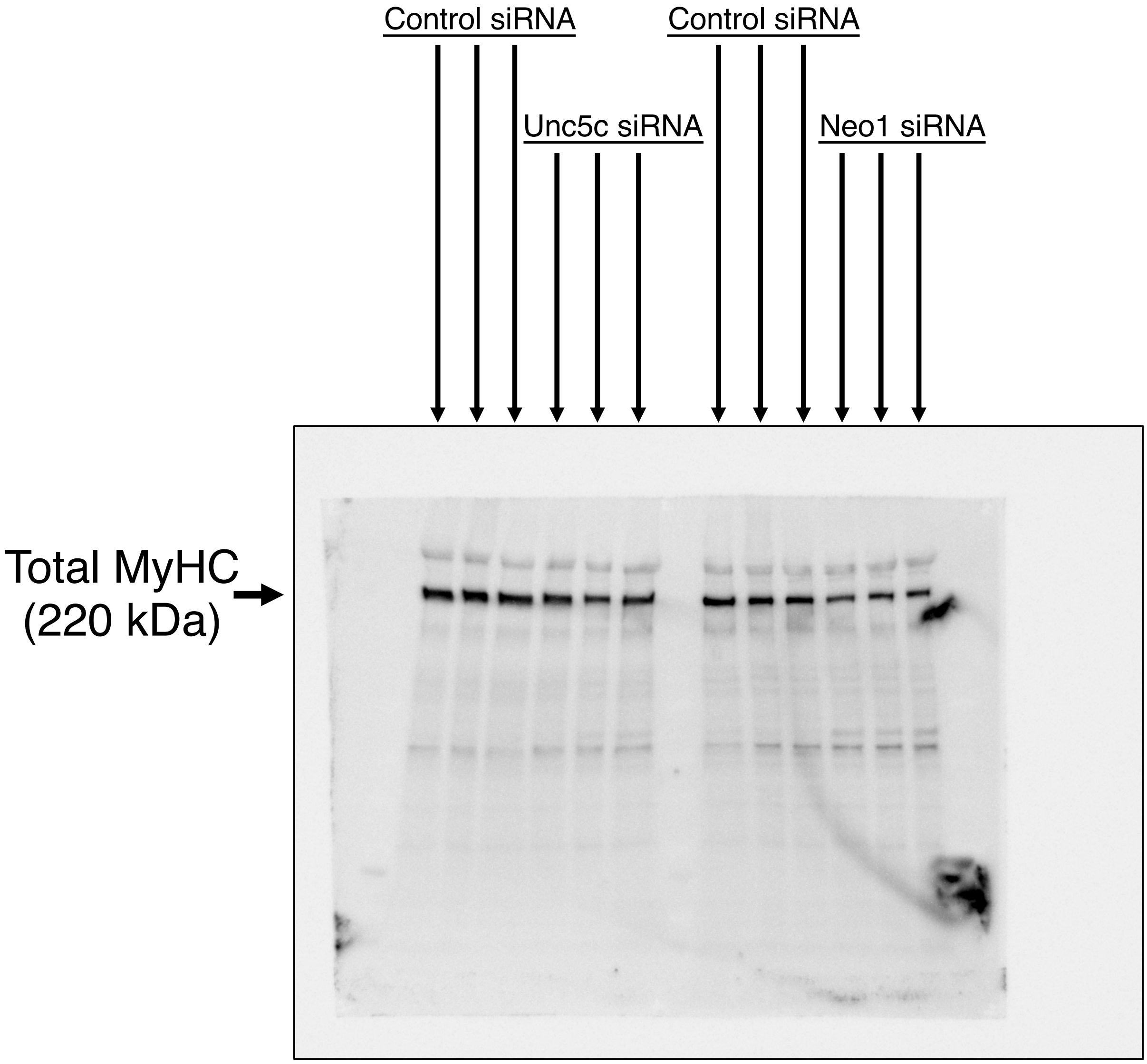

Fig. S2I

< Slow MyHC >

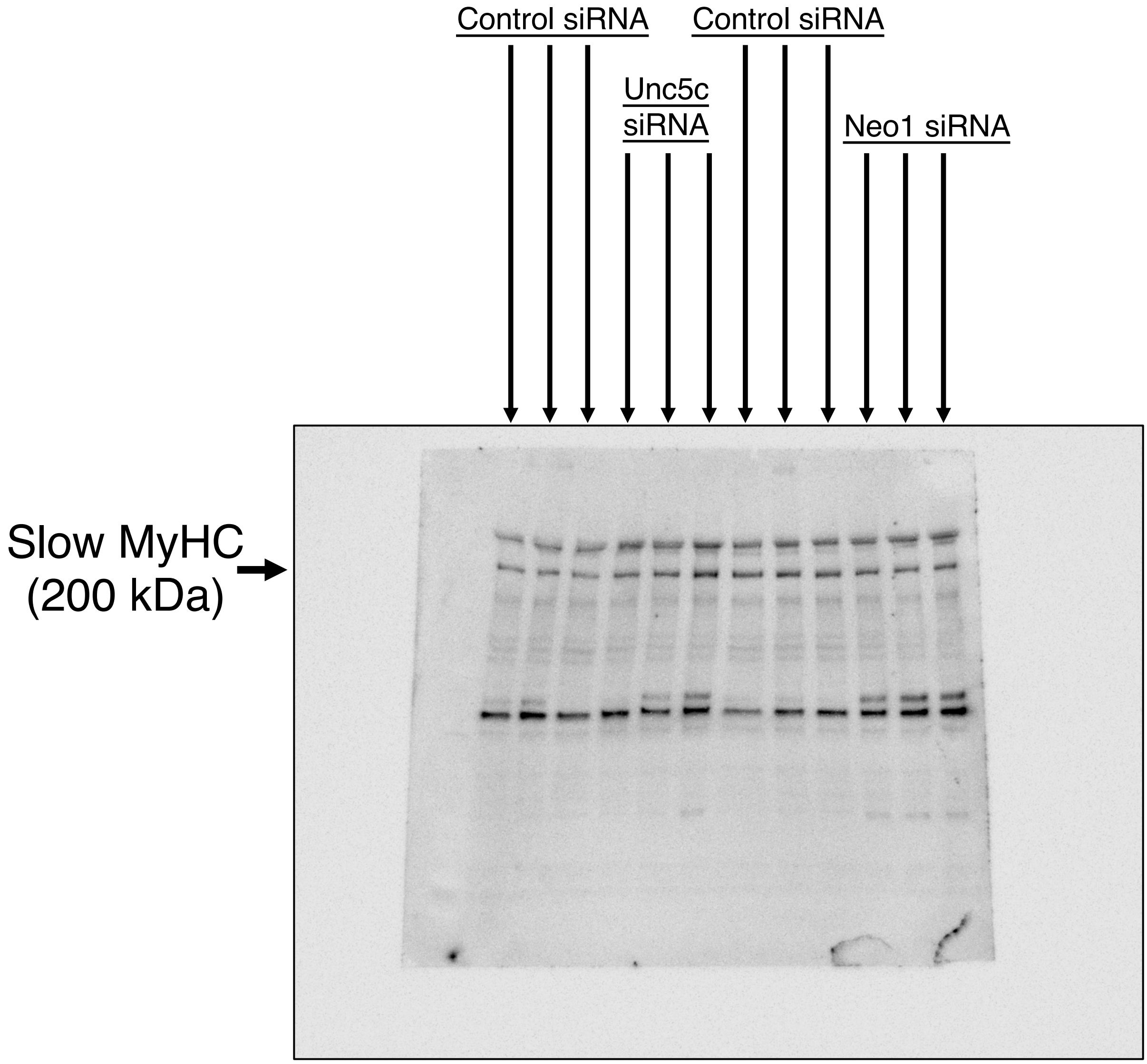

Fig. S2J

< Fast MyHC >

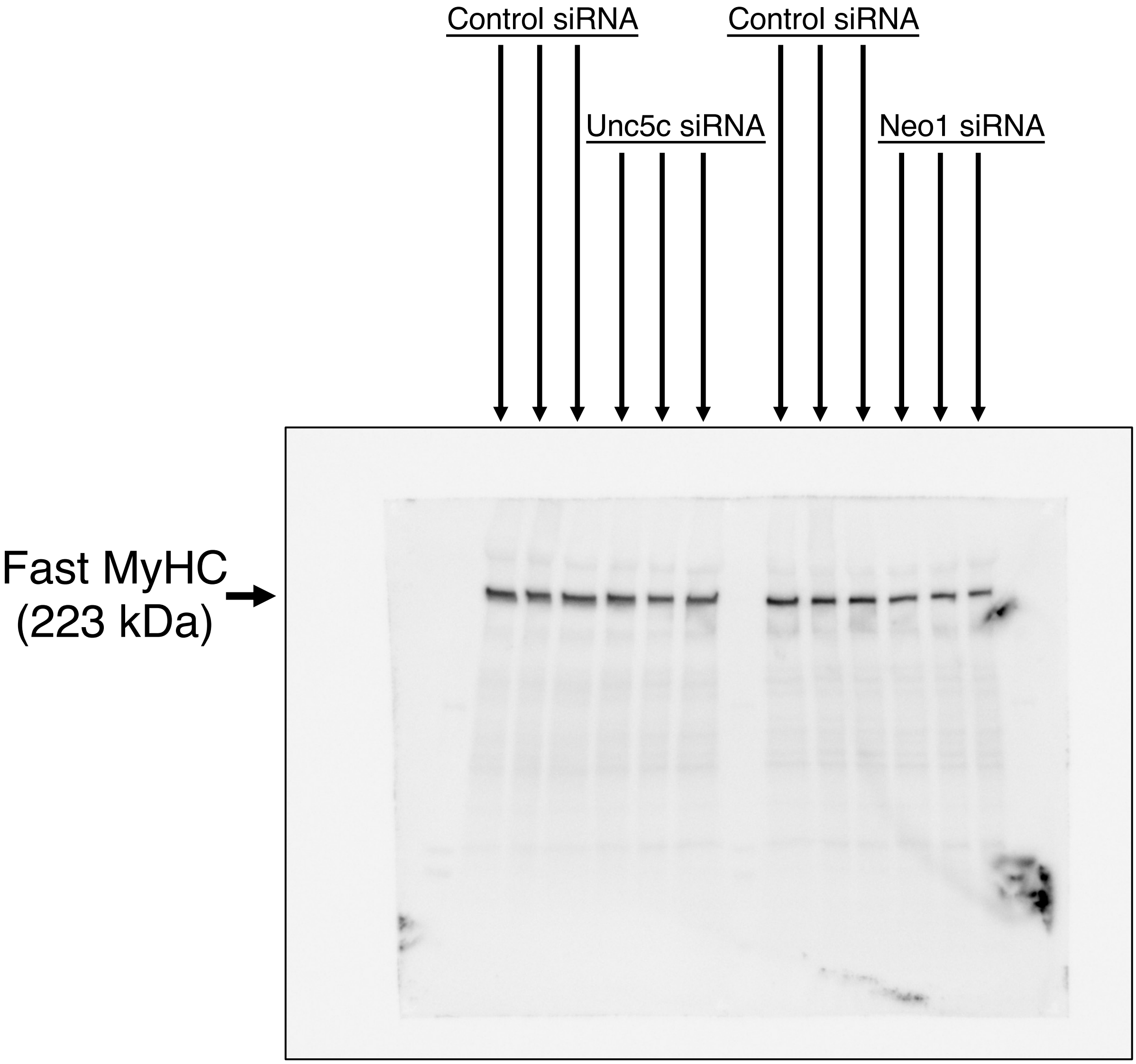

Fig. S2K

< MyHC IIa >

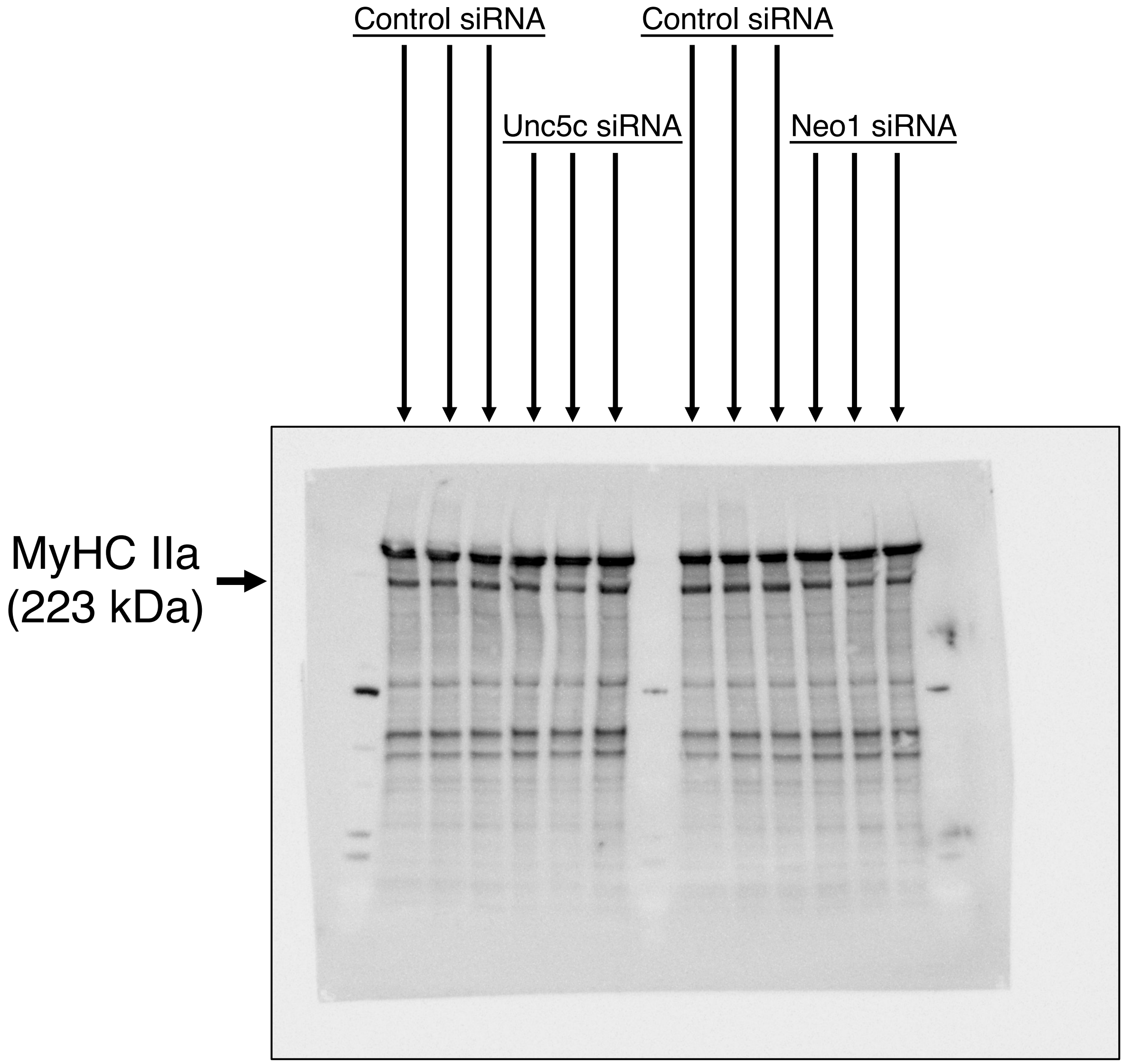

Fig. S2L

< MyHC IIx >

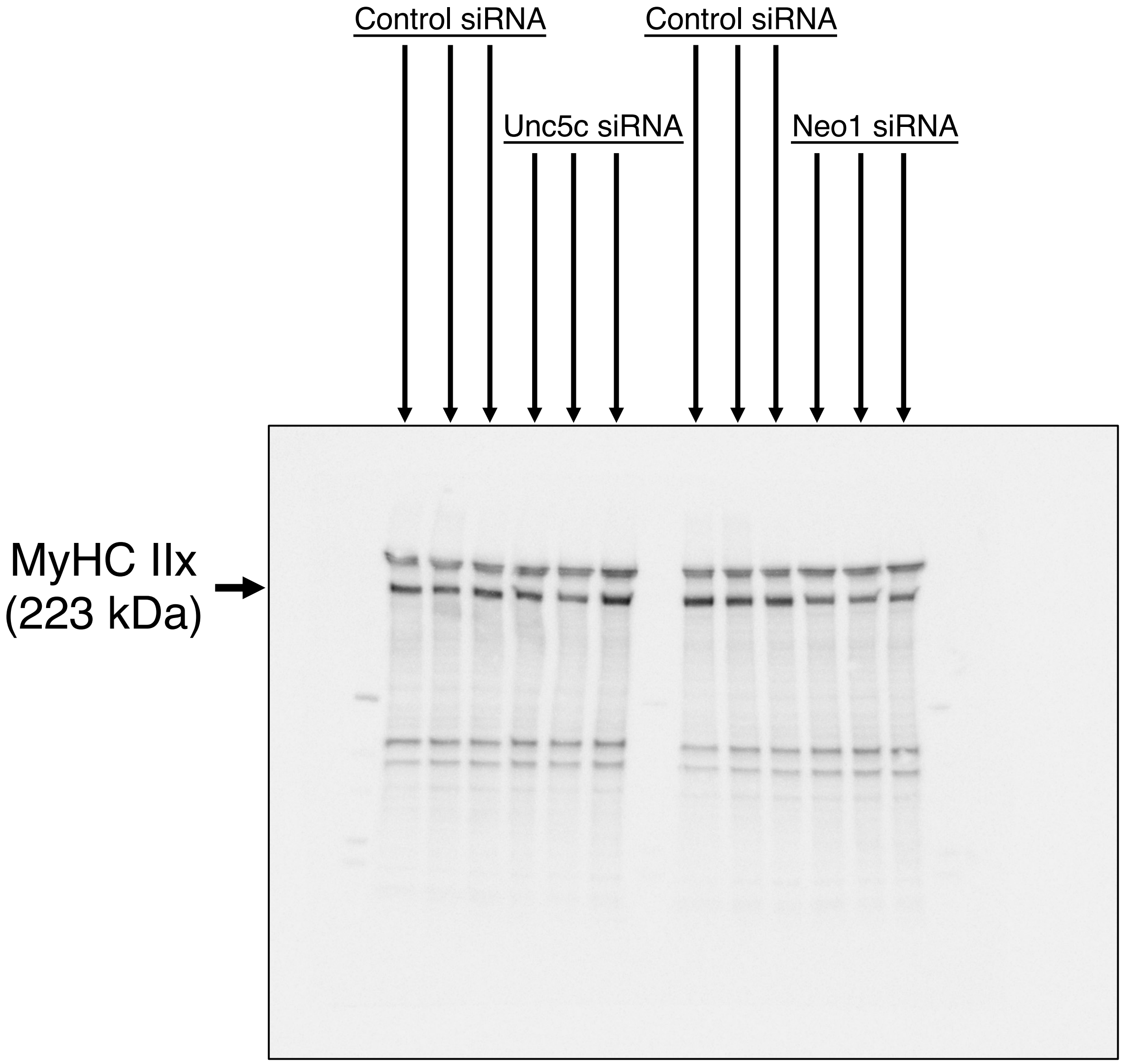

Fig. S2M

< MyHC IIb >

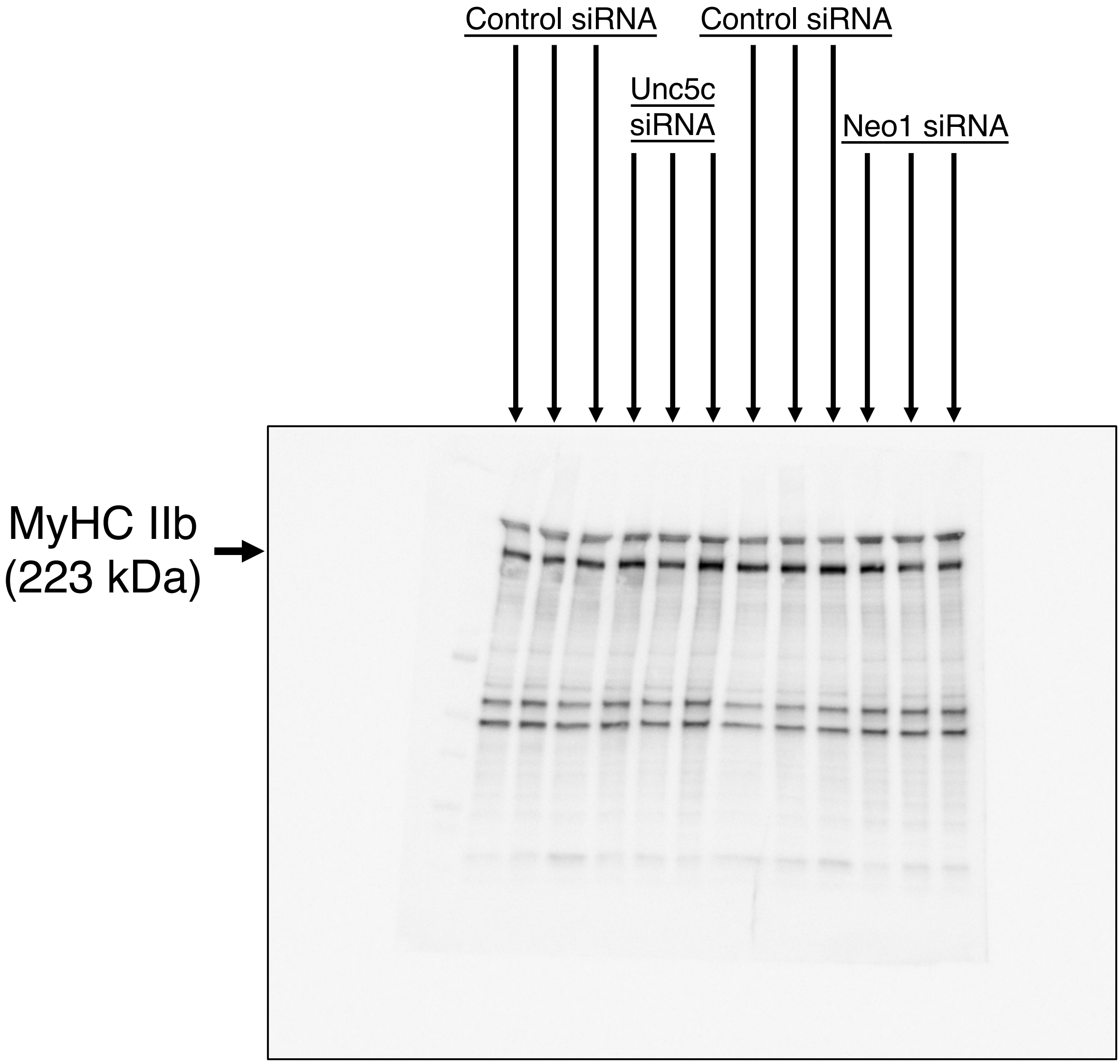

Fig. S2N

< α-tubulin >

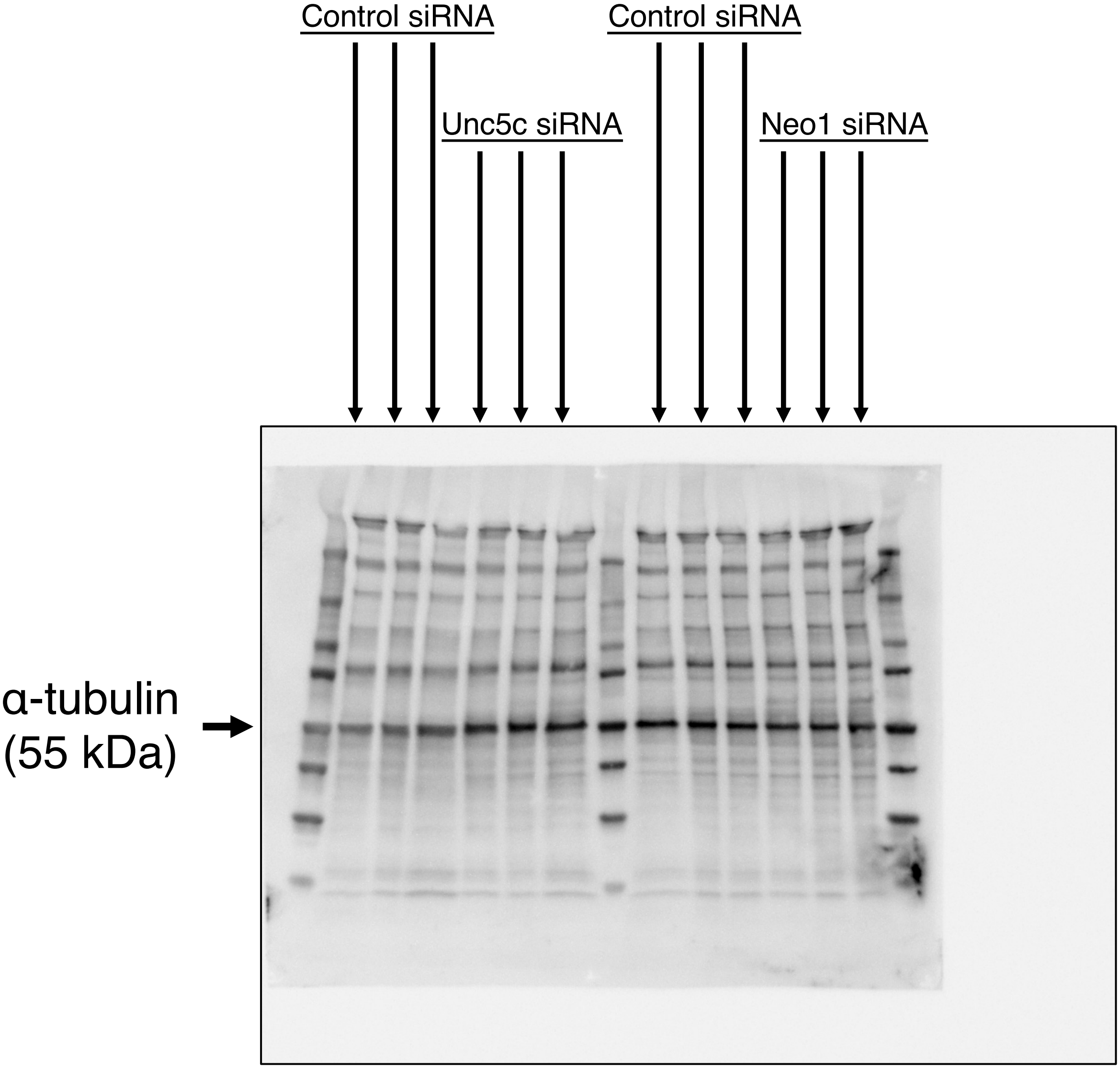

Supplement: Supplementary file 2 — Data S2. Supporting Information. [file PHY2-14-e70788-s001.pdf]
